# Functional genomics atlas of synovial fibroblasts defining rheumatoid arthritis heritability

**DOI:** 10.1186/s13059-021-02460-6

**Published:** 2021-08-25

**Authors:** Xiangyu Ge, Mojca Frank-Bertoncelj, Kerstin Klein, Amanda McGovern, Tadeja Kuret, Miranda Houtman, Blaž Burja, Raphael Micheroli, Chenfu Shi, Miriam Marks, Andrew Filer, Christopher D. Buckley, Gisela Orozco, Oliver Distler, Andrew P. Morris, Paul Martin, Stephen Eyre, Caroline Ospelt

**Affiliations:** 1 Centre for Genetics and Genomics Versus Arthritis, Centre for Musculoskeletal Research, School of Biological Sciences, Faculty of Biology, Medicine and Health, The University of Manchester, Manchester, UK; 2 Department of Rheumatology, Center of Experimental Rheumatology, University Hospital Zurich, University of Zurich, Zurich, Switzerland; 3 Department of Rheumatology, University Medical Centre, Ljubljana, Slovenia; 4 Schulthess Klinik, Zurich, Switzerland; 5 Institute of Inflammation and Ageing, University of Birmingham, Birmingham, UK; 6 NIHR Birmingham Biomedical Research Centre, University Hospitals Birmingham NHS Foundation Trust, University of Birmingham, Birmingham, UK; 7 Kennedy Institute of Rheumatology, University of Oxford, Roosevelt Drive, Headington, Oxford, UK; 8 NIHR Manchester Biomedical Research Centre, Manchester Academic Health Science Centre, Manchester University Foundation Trust, Manchester, UK; 9 The Lydia Becker Institute of Immunology and Inflammation, Faculty of Biology, Medicine and Health, University of Manchester, Manchester, UK

**Keywords:** Functional genomics, Stromal cells, Rheumatoid arthritis, Fibroblast-like synoviocytes

## Abstract

**Background:**

Genome-wide association studies have reported more than 100 risk loci for rheumatoid arthritis (RA). These loci are shown to be enriched in immune cell-specific enhancers, but the analysis so far has excluded stromal cells, such as synovial fibroblasts (FLS), despite their crucial involvement in the pathogenesis of RA. Here we integrate DNA architecture, 3D chromatin interactions, DNA accessibility, and gene expression in FLS, B cells, and T cells with genetic fine mapping of RA loci.

**Results:**

We identify putative causal variants, enhancers, genes, and cell types for 30–60% of RA loci and demonstrate that FLS account for up to 24% of RA heritability. TNF stimulation of FLS alters the organization of topologically associating domains, chromatin state, and the expression of putative causal genes such as TNFAIP3 and IFNAR1. Several putative causal genes constitute RA-relevant functional networks in FLS with roles in cellular proliferation and activation. Finally, we demonstrate that risk variants can have joint-specific effects on target gene expression in RA FLS, which may contribute to the development of the characteristic pattern of joint involvement in RA.

**Conclusion:**

Overall, our research provides the first direct evidence for a causal role of FLS in the genetic susceptibility for RA accounting for up to a quarter of RA heritability.

**Supplementary Information:**

The online version contains supplementary material available at 10.1186/s13059-021-02460-6.

## Background

A major challenge of the post-genome-wide association study (GWAS) era is to decipher the functional consequences of genetic risk variants in individual cell types and their contribution to the development of polygenic diseases. The identification of the cell types and conditions in which genetic risk variants are effective is an essential prerequisite for achieving this goal. Rheumatoid arthritis (RA) is a symmetric inflammatory and destructive autoimmune arthritis with a complex genetic basis. RA affects 0.5–1% of the world population and leads to disability, high morbidity burden, and premature mortality [1]. GWAS have identified over 100 loci for RA susceptibility [2]. Genetic risk variants at the majority of these loci do not map to the exons of protein coding genes. Potential gene regulatory functions of these noncoding genetic risk variants have been investigated in immune cells based on genome-wide mapping of epigenetic modifications [3], chromatin interactions [4], correlation with variation in gene expression (eQTLs) [5], or linear proximity to coding genes in DNA sequence [2]. These studies have demonstrated an enrichment of RA genetic risk variants in immune cell enhancers [3], but omitted the analysis of synovial fibroblasts or fibroblast-like synoviocytes (FLS), the resident stromal cells of the joints, even though they are responsible for the production of many immune-related cytokines and chemokines [6, 7].

In addition to immune cells, FLS play a decisive role in the pathogenesis of RA and are essential for the maintenance of normal joint functions. FLS from different joints have different epigenomes, transcriptomes, and functions, which may contribute to the characteristic pattern of joint involvement in different types of arthritis [8, 9]. FLS substantially contribute to joint inflammation and destruction in RA [10]. RA FLS have an activated phenotype characterized by resistance to apoptosis, increased proliferation, secretion of matrix-degrading enzymes, and production of cytokines and chemokines that promote immune cell differentiation and survival. However, the cause of the activation of FLS in RA is unknown and it is unclear whether this activation leads to or is a consequence of the disease. Defining the contribution of FLS to the heritability of RA will provide essential insights into this question.

For the first time, we have comprehensively mapped RA genetic risk variants to active regulatory DNA elements in FLS. We generated multidimensional epigenetic data in primary FLS, isolated from patients, to create a detailed outline of their chromatin landscape. We conducted genetic fine mapping of RA loci by computing sets of credible single-nucleotide polymorphisms (SNPs) driving GWAS signals. We integrated the credible SNP sets and chromatin datasets to provide evidence that RA risk variants can be functionally relevant in FLS. We used chromatin conformation data to determine enhancer–promoter interactions between risk variants in noncoding DNA regulatory regions of FLS and their target genes. Furthermore, we assessed the influence of the pro-inflammatory cytokine tumor necrosis factor (TNF) on these interactions, chromatin accessibility, and gene expression in FLS. We combined FLS data with published data of human tissues and cells [4, 11, 12] to identify putative causal SNPs, enhancers, genes, and cell types for RA risk loci. Finally, we functionally verified enhancer-promoter interactions by CRISPR-Cas technology and showed transcriptional effects of fine-mapped risk variants in FLS samples from RA patients.

## Results

### Integration of epigenetic datasets to define the chromatin landscape of FLS

**Fig. 1:**
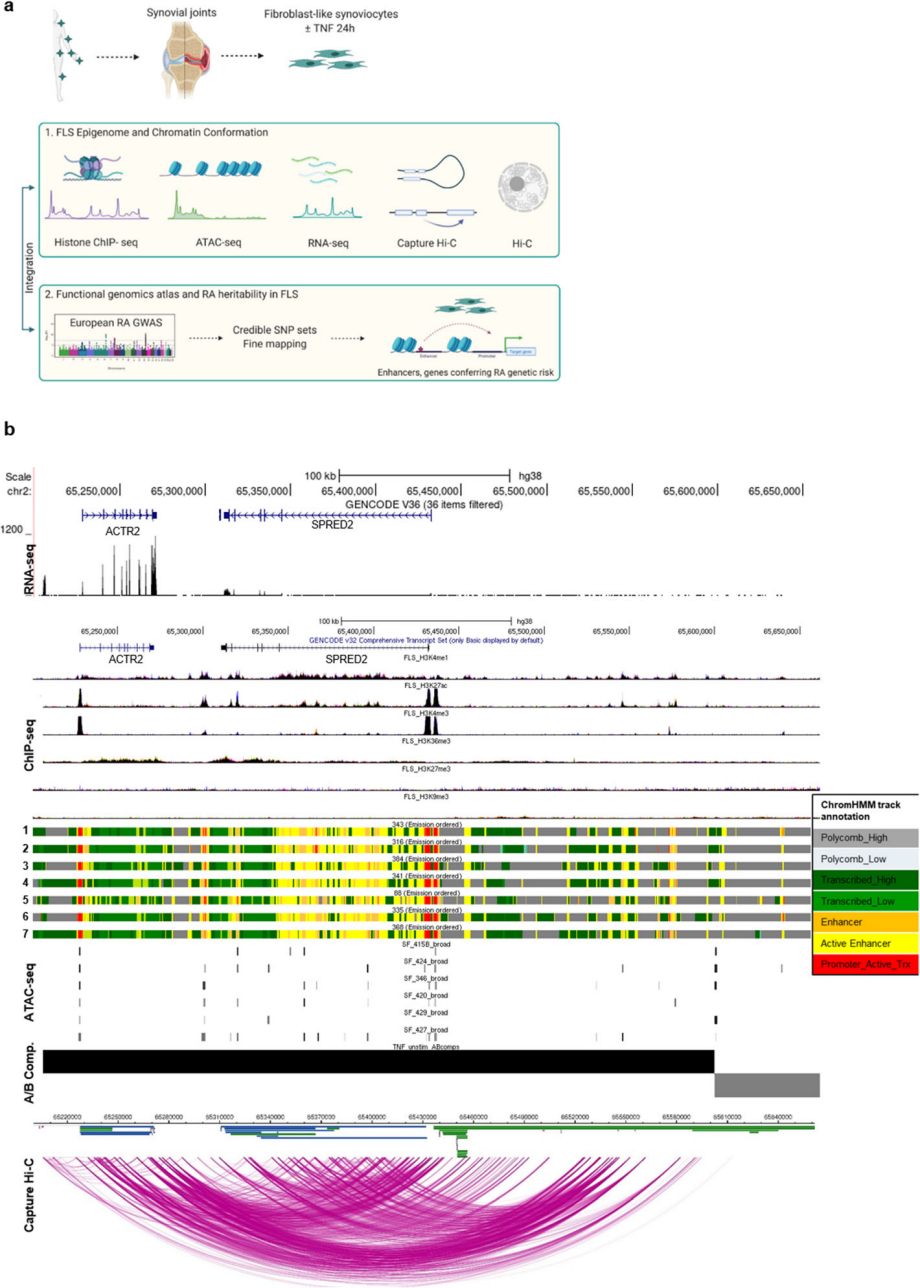
Epigenomic and 3D chromatin atlas of human FLS. **a** Schematic representation of the workflow to comprehensively annotate the transcriptome, epigenome, and chromatin structure of FLS and define their contribution to RA heritability. This figure was created using BioRender. **b** The SPRED2 locus as an example genomic region demonstrating the annotation of epigenetic states and chromatin architecture in unstimulated FLS. Shown are from top to bottom, one exemplary RNA-seq track, ChIP-seq peaks (H3K4me1, H3K27ac, H3K4me3, H3K36me3, H3K27me3, H3K9me3), ChromHMM annotation in 7 different FLS lines (1: OA hand FLS, 2: RA hand FLS, 3: OA shoulder FLS, 4: RA shoulder FLS, 5: healthy knee FLS, 6: OA knee FLS, 7: RA knee FLS), ATAC-seq peaks in 6 different RA FLS lines, A/B compartments (black bar open chromatin, gray bar closed chromatin), chromatin interactions (Capture HiC)

As a first step in our analysis, we generated diverse epigenetic and transcriptomic datasets from our primary FLS samples (Additional file 1: Table S1): chromatin immunoprecipitation sequencing (ChIP-seq) for six histone marks (H3K4me3, H3K4me1, H3K27me3, H3K36me3, H3K27ac, H3K9me3), Assay for Transposase-Accessible Chromatin sequencing (ATAC-seq), cap analysis gene expression sequencing (CAGE-seq), chromatin conformation analysis (HiC, Capture HiC), and RNA sequencing (RNA-seq) (Additional file 2: Table S2, quality control metrics in additional file 3: Dataset S1, details in Online methods). For Capture HiC (CHiC), prey fragments containing previously reported lead SNPs at RA loci were used [2] (details in Online methods). We integrated these datasets and assigned 18 pre-trained chromatin states to the genome of FLS using ChromHMM [13]. We identified A/B compartments and Topologically Associating Domains (TADs) and determined significant chromatin interactions. Finally, we incorporated RNA-seq data from FLS. These analyses provided a comprehensive annotation of the epigenome and transcriptome of FLS (Fig. 1a, b).

**Fig. 2:**
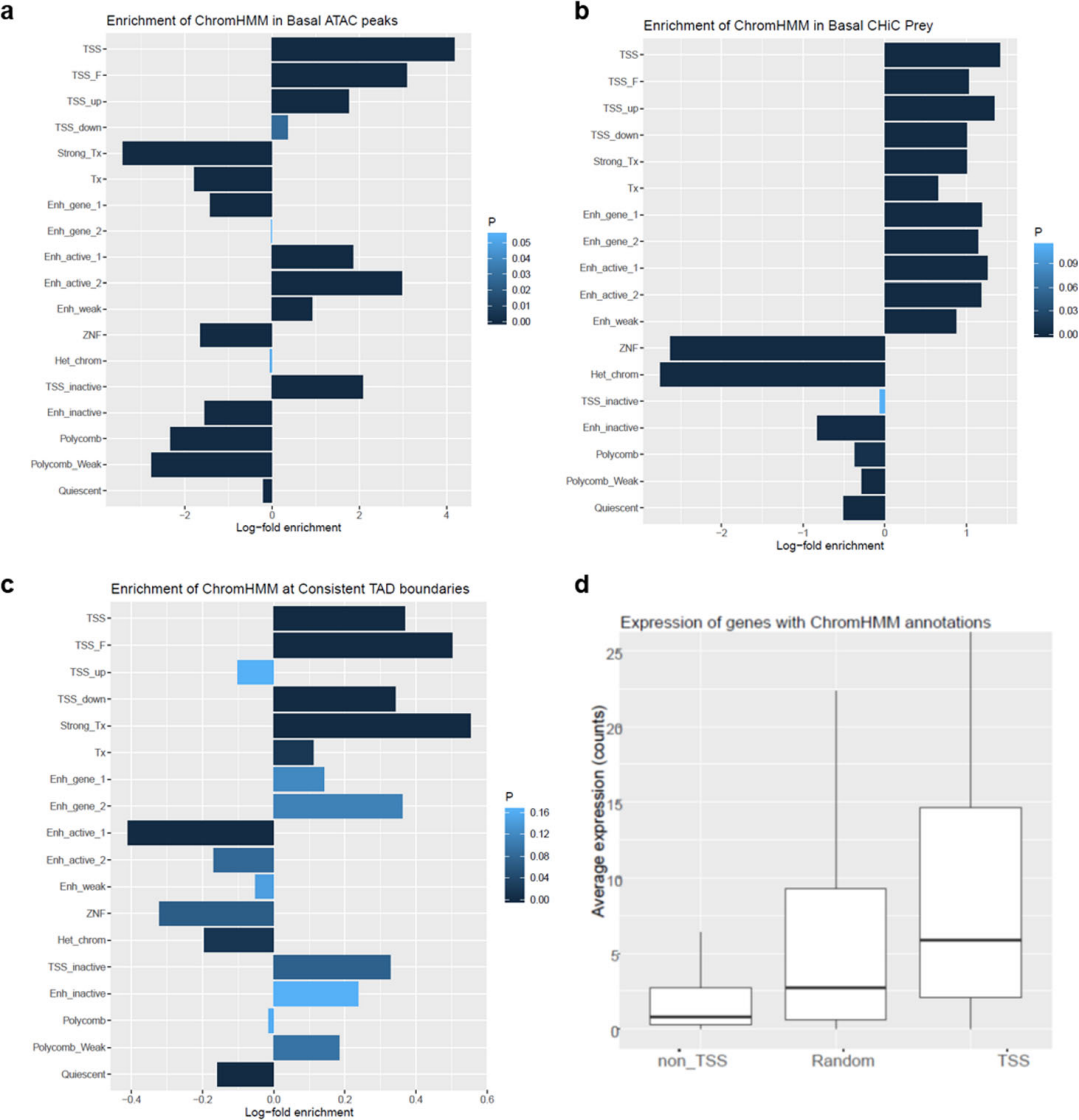
Cross validation of generated datasets defining the chromatin landscape of FLS. **a** Log fold change enrichment of chromatin states as defined by ChromHMM in open chromatin regions as identified by ATAC-seq. **b** Log fold change enrichment of chromatin states as defined by ChromHMM in prey fragments of Capture HiC measurements. **c** Log fold change enrichment of chromatin states as defined by ChomHMM in consistent TAD boundaries. **d** Basal average expression of genes (RNA-seq counts) across non-TSS, TSS, and random ChromHMM annotations. TSS = transcription start site, TSS_F = flanking TSS; TSS_up = upstream TSS; TSS_down = downstream TSS; Tx = Transcription; Enh_gene = enhancer genic; ZNF = zinc finger; Het_chrom = heterochromatin

We cross-validated the individual datasets to confirm the quality of the generated FLS data. As expected, open chromatin regions showed high enrichment of promoters (transcription start sites [TSS]) and active enhancers (Fig. 2a). CHiC interactions were enriched for promoters (TSS), sites of transcription, and enhancers (Fig. 2b). At TAD boundaries, transcription and promoter states were enriched (Fig. 2c). Basal gene expression was highest in active TSS (Fig. 2d). Taken together, these analyses validated that we accurately captured chromatin states and chromatin interactions in FLS and that we have generated a comprehensive epigenetic and transcriptomic map of FLS genomes.

### TNF induces changes in chromatin organization that correspond to altered gene expression in stimulated FLS

To explore the effect of a pro-inflammatory environment on the chromatin landscape and transcriptional regulation of FLS, we performed HiC, CHiC, ATAC-seq, and RNA-seq experiments in FLS with and without stimulation with TNF (Additional file 2: Table S2).

We first computed changes in A/B compartments, which are large, cell-type-specific organizational units of the genome, associated with chromatin activity (A = open chromatin, B = closed chromatin) [14]. 94.8% of A and 95.7% of B compartments were consistent between basal and stimulated FLS. One of the genomic regions that changed from inactive to active after TNF stimulation contained RA-associated variants that interact with the *TNFAIP3* gene. Small changes in A/B compartments after stimulation are expected, as A/B compartments infer chromatin activity at DNA segments in low resolution.

**Fig. 3:**
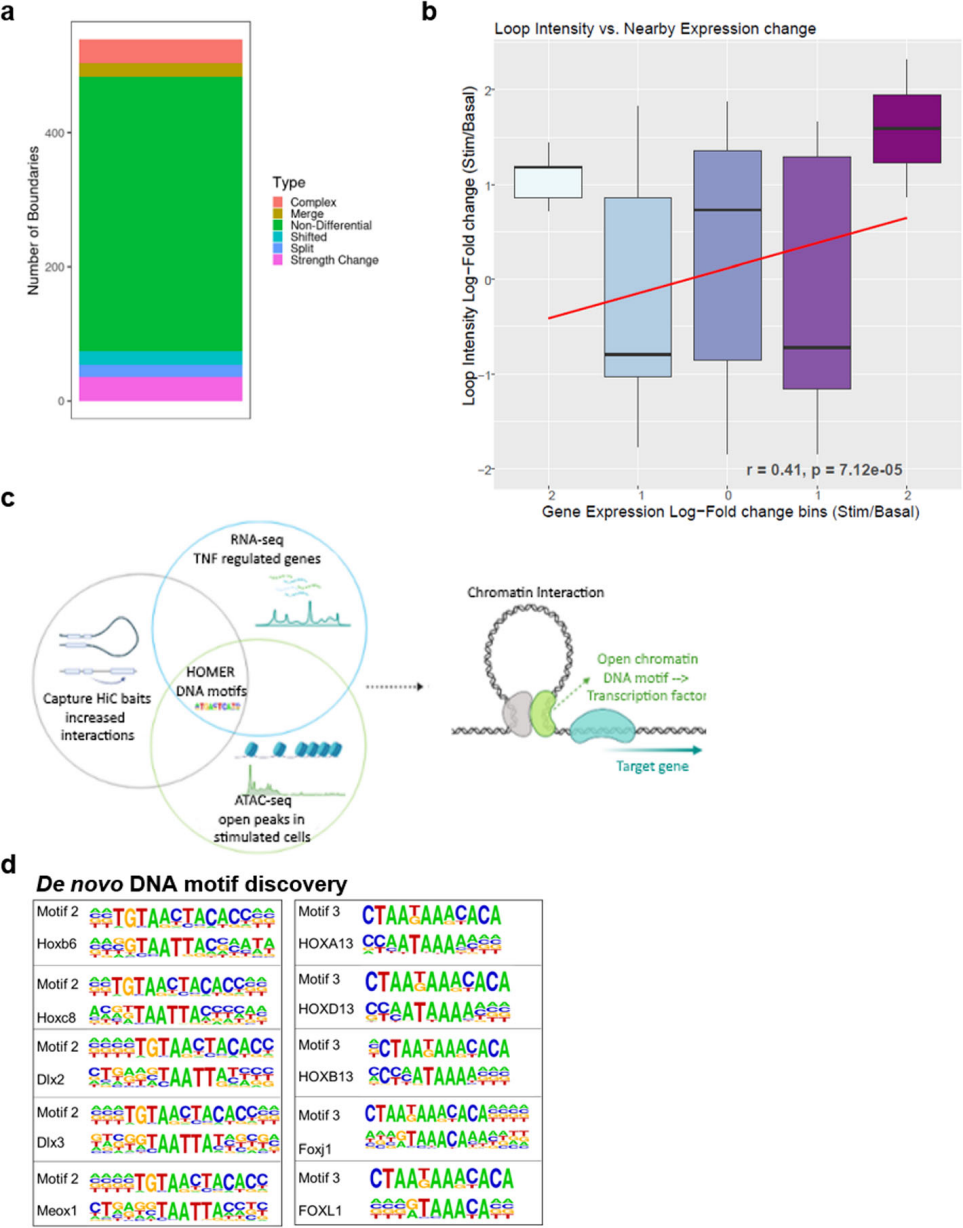
Effect of TNF stimulation on the chromatin landscape in FLS. **a** Comparison of TADs boundaries between basal and TNF-stimulated FLS by TADCompare. Number of non-differential (green) and differential TAD boundaries is shown. Differential TAD boundaries are classified as boundary position changes (complex, merge, shifted, split) or strength change (differential boundary magnitude). Complex, merged, and split boundary changes represent the most disruptive changes of the 3D structure of the genome. **b** Pearson correlation of the loop intensity as determined by CHiC with change in the expression of nearby genes (log fold change). Negative numbers in the *x*-axis indicate downregulation, positive numbers upregulation of gene expression. **c** Graphical representation of the RNA-seq, ATAC-seq, and CHiC data integration to identify transcription factor binding sites in TNF-stimulated FLS. This figure was created using BioRender. **d** De novo DNA motif discovery identified two motifs (motif 2 and motif 3) with high similarity to the binding sites of homeobox (TAATTA) and forkhead box transcription factors (TAAA) in the dataset with TNF-repressed genes.

To increase the resolution, we used TADcompare [15] to explore the influence of TNF on the organization of TADs in FLS. Genes within the same TAD tend to be co-regulated and gene promoters and enhancers often interact within the same TAD [16]. Between our conditions, we identified an average of 4116 TAD boundaries in FLS samples. While 79% of TAD boundaries were unchanged between basal and stimulatory conditions, 21% of differential TAD boundaries exhibited a change in position or strength (Fig. 3a).

By analyzing CHiC data (details in Online methods), we observed around 800 quantitatively differentially interacting regions between basal and stimulated FLS. The intensity of the differential interactions between the regions correlated with the fold change of expression of the interacting genes (Fig. 3b). Notably, interaction strength increased after stimulation for genes with differential expression, irrespective of whether expression increased or decreased after stimulation, thereby suggesting that chromatin interactions influence activating and repressive TNF transcriptional responses in FLS (Fig. 3b).

To further explore the regulation of gene transcription after TNF stimulation, we focused on CHiC baits and prey that exhibited increased interaction strength with genes regulated after stimulation. We overlapped these regions with open chromatin peaks in stimulated cells. We then used Hypergeometric Optimization of Motif EnRichment (HOMER) to detect known transcription factor binding sites (TFBS) or DNA motifs with high similarity to known TFBS (de novo DNA motif discovery), that were overrepresented at the sites with open chromatin, increased chromatin interactions, and differential gene expression (Fig. 3c).

Enrichment analysis of known TFBS in open chromatin identified TPA response elements (TREs; TGA(G/C)TCA) as the most enriched motif in the data sets with increased as well as decreased gene expression (Additional file 4: Dataset S2). TPA response elements serve as canonical binding sites for the subunits of the Activator Protein-1 (AP-1) transcription factor. Open chromatin sites with increased CHiC interactions, but decreased gene expression in stimulated FLS were additionally enriched for BACH2 (broad-complex-tramtrack-bric-a-brac and Cap'n'collar homology 2) binding sites (Additional file 4: Dataset S2). De novo DNA motif discovery in the dataset with decreased levels of gene expression after TNF stimulation showed enrichment for two DNA motifs with high similarity to binding sites for several developmental transcription factors from homeobox and forkhead box protein families (Fig. 3d).

In summary, by combining CHiC, ATAC-seq, and RNA-seq analyses, we showed that the FLS genome exhibits changes in 3D structure upon TNF stimulation. We confirmed the activating and repressive actions of AP-1 in regulating the TNF response of FLS and we suggest that developmental transcription factors can serve as potential novel repressors of transcriptional response to TNF in FLS.

### FLS and immune cells are drivers of RA heritability

**Fig. 4:**
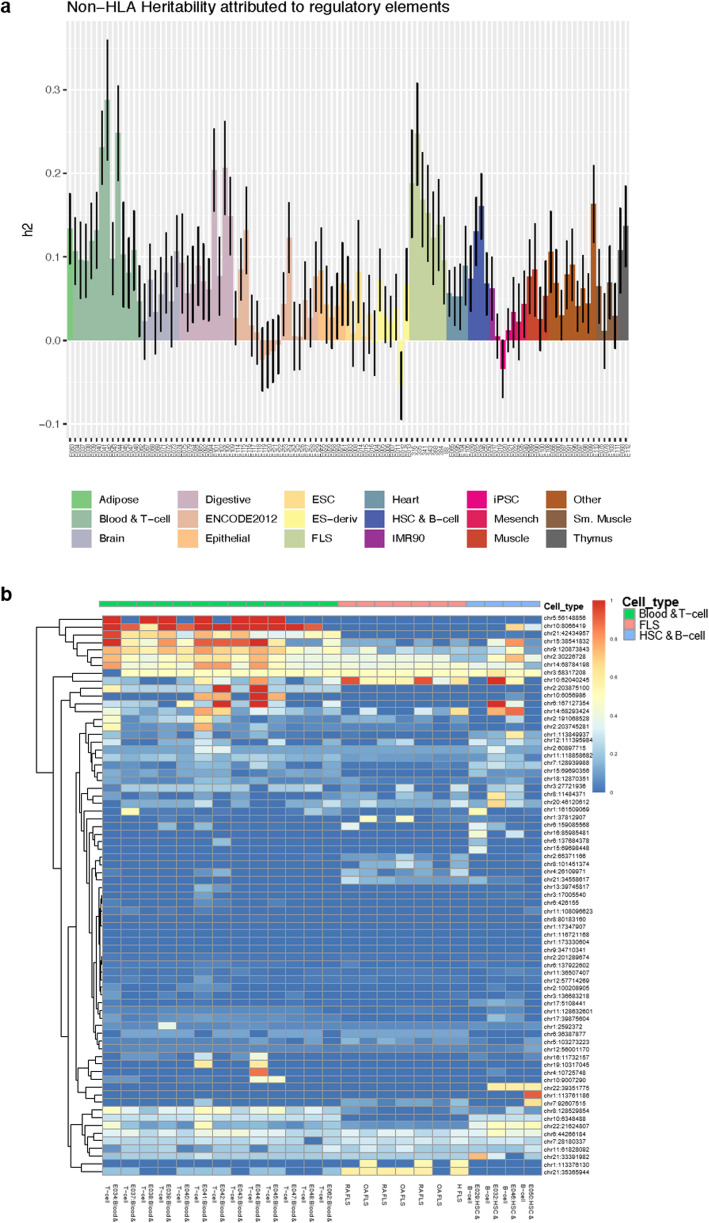
Heritability and causal SNPs in FLS. **a** Partitioned heritability (h2) of RA attributed to active regions in each sample of FLS (*n* = 7) and 111 available Roadmap cell types/tissues (Epigenomics Mapping Roadmap Consortium [11]). **b** The sum of posterior probability overlapping active DNA regulatory elements across blood and T cell samples (Epigenomics Mapping Roadmap Consortium; green bar), FLS samples (red bar), human stem cells (HCS), and B cells (Epigenomics Mapping Roadmap Consortium; blue bar) at each of the 73 sites. Active DNA regulatory elements were defined as the union of H3K4me3, H3K4me1, and H3K27ac marks

We used the generated knowledge on regulatory DNA elements in FLS to quantify the heritability of RA that can be attributed to active regulatory DNA elements in FLS. We considered RA risk loci attaining genome-wide significance (*p* < 5 × 10^−8^) in the European ancestry component of the largest published trans-ethnic RA GWAS meta-analysis [2] and computed the partitioned heritability [17] in FLS and other cell types (HLA regions excluded; details in “Methods” section). Epigenetic data for non-FLS cell types were acquired from published datasets [11]. We defined active regulatory elements of the genome as the union of H3K4me1, H3K4me3, and H3K27ac peaks, as these histone modifications are associated with transcriptional activity and enhancer/promoter elements. With this approach, we estimated that 12–24% of the non-HLA RA heritability can be attributed to the active DNA regulatory elements in FLS samples (Fig. 4a). This analysis showed that both immune cells and FLS mediate the effects of association signals and contribute notably to the heritability of RA.

### Epigenetic annotation of fine-mapped SNPs in immune cells and FLS refines the putative causal credible set SNPs for more than 30% of the RA risk loci

We then aimed to further characterize the RA SNPs in active DNA regulatory regions in FLS (Additional file 5: Fig S1). We first used approximate conditional analyses implemented in genome-wide complex trait analysis (GCTA) [18] to dissect the previously identified RA risk loci [2]. Where lead SNPs at genomic loci mapped within 1 Mb of each other, the loci were merged (Additional file 6: Table S3). We identified 73 distinct signals of association with RA at locus-wide significance (*p* < 10^−5^), with each signal being potentially driven by different underlying causal variants (Additional file 7: Table S4). For each signal, we performed fine mapping to derive credible SNP sets that together account for ≥ 99% of the posterior probability of causality for the RA association. Across all 73 signals, the RA credible SNP sets included a total of 8787 variants, of which 2654 variants had posterior probability of causality > 0.01% (Additional file 8: Table S5; Additional file 5: Fig S1).

We then overlapped the 2654 RA credible SNPs with the FLS epigenome and identified 274 SNPs within 23 associated signals mapping to active DNA regulatory elements in FLS (Fig. 4b). We also calculated the total posterior probability across the credible SNP sets found within active DNA regulatory elements for 111 primary cell types and tissues, whose epigenomes were published by the Roadmap Epigenomics Mapping Consortium [11] (Fig. 4b, Additional file 5: Fig S2). As expected, several credible SNP sets exhibited high posterior probability in active DNA regulatory elements from B and T cells (*n* = 35 signals), of which some (*n* = 14 signals) also overlapped active DNA regulatory regions in FLS (Fig. 4b, Additional file 8: Table S5 column R). Intriguingly, we identified several credible SNP sets that were active in FLS only, but not in B and T cells (*n* = 9 signals; Fig. 4b, Additional file 8: Table S5 column R).

Based on our genetic fine-mapping analysis, we assigned the association signals to three categories (Additional file 8: Table S5 column K, Table 1). First, well characterized signals (“category 1,” *n* = 19), where the credible set included ten or fewer SNPs or ≤ 3 SNPs contributing > 80% of the posterior probability of causality. Second, localized associated signals (“category 2”, *n* = 26), where the credible set included ≤ 20 SNPs with similar low posterior probabilities (10–20%) or the lead credible SNP accounted for > 20% of the posterior probability. Third, poorly characterized signals (“category 3,” *n* = 28), where genetic fine mapping was largely ineffective, resulting in large (> 20) credible SNP sets with equally negligible posterior probabilities (< 5%) (Additional file 8: Table S5 column K, Table 1).

**Table 1:** Identified risk loci for RA with less than three credible SNPs and the cell type in which they are active

Locus	Chr	Number of credible SNPs (> posterior prior 0.001)	Posterior probability top SNP	Total posterior probability top 3 SNPs	SNP category	Number of SNPs in 30% of FLS enhancers (posterior > 0.001)	Number of SNPs in 30% of T cell enhancers (posterior > 0.001)	Number of SNPs in 30% of B cell enhancers (posterior> 0.001)	FLS, T, B, ALL, NONE (based on posteriors of SNPs in enhancers)	Top posterior rs numbers
4	chr1	10	0.53	0.94	1	1	0	0	FLS	rs4839319, rs4839318, rs77227025
5	chr1	2	0.91	0.99	1	0	0	0	B CELL	rs6679677, rs2476601
7	chr1	10	0.25	0.62	1	0	0	0	NONE	rs624988, rs771587, rs12137270, rs12405671, rs11586238
10	chr2	9	0.21	0.59	1	2	3	2	ALL	rs10175798, rs10173253, rs906868, rs7579944, rs1355208
17	chr2	4	0.43	0.92	1	0	2	0	T CELL	rs231724, rs231723, rs231775
20	chr3	13	0.34	0.82	1	4	2	3	ALL	rs73081554, rs185407974, rs180977001, rs35677470, rs114584537
24	chr5	1	1	1	1	0	1	0	T CELL	rs7731626
29	chr6	8	0.23	0.67	1	0	0	0	NONE	rs17264332, rs11757201, rs6920220, rs6927172
30	chr6	15	0.42	0.86	1	2	5	3	NONE	rs58721818, rs61117627
32	chr6	9	0.32	0.71	1	0	5	0	T CELL, B CELL	rs1571878, rs3093017, rs10946216
40	chr9	3	0.37	0.98	1	0	0	0	NONE	rs11574914, rs2812378, rs10972201
42	chr10	6	0.73	0.87	1	0	0	0	T CELL	rs706778, rs10795791
44	chr10	10	0.21	0.49	1	0	10	0	T CELL	rs537544, rs568727, rs570613 , rs570730, rs7897792
46	chr10	10	0.37	0.73	1	9	1	0	ALL	rs12764378, rs71508903, rs77509998
60	chr15	5	0.26	0.74	1	0	0	0	NONE	rs919053, rs8026898, rs7170107, rs16953656, rs8043362
62	chr16	9	0.29	0.82	1	0	0	0	B CELL	rs13330176, rs2139492, rs2139493
66	chr19	2	0.6	1	1	0	0	0	NONE	rs74956615, rs34536443
70	chr21	19	0.56	0.93	1	3	1	0	FLS	rs8133843, rs8129030, rs9979383
73	chr22	9	0.29	0.74	1	0	0	2	B CELL	rs2069235, rs909685, rs9611155
1	chr1	81	0.29	0.38	2	1	1	1	NONE	rs187786174, rs60733400, rs876938
2	chr1	13	0.29	0.52	2	0	0	0	NONE	rs2240336, rs12737739, rs13375202
3	chr1	36	0.43	0.51	2	1	0	0	FLS	rs28411352, rs28489009, rs2306627
6	chr1	29	0.22	0.42	2	0	2	4	B CELL	rs1217404, rs2476604, rs1217420
11	chr2	13	0.18	0.4	2	1	1	1	ALL	rs34695944, rs56095903, rs67574266, rs13031237, rs13031721
13	chr2	17	0.27	0.63	2	0	0	1	NONE	rs9653442, rs6712515, rs11676922, rs1160542, rs10865035
18	chr3	63	0.27	0.55	2	0	0	0	NONE	rs4452313, rs4416363 , rs7617779
19	chr3	11	0.21	0.48	2	1	1	0	T CELL, B CELL	rs9310852, rs4680838, rs9880772, rs1353286
22	chr4	49	0.2	0.29	2	0	0	0	NONE	rs7660626, rs13142500, rs6831973
31	chr6	13	0.17	0.49	2	0	2	2	B CELL	rs2451258, rs2485363, rs654690 , rs1994564, rs212389
33	chr7	47	0.24	0.47	2	3	1	2	ALL	rs186735625, rs57585717, rs2158624
36	chr8	20	0.08	0.23	2	3	1	0	FLS, B CELL	rs2736337
38	chr8	11	0.15	0.39	2	1	0	0	FLS	rs678347, rs507201, rs657425
43	chr10	17	0.27	0.45	2	0	3	2	T CELL, B CELL	rs947474, rs10796038, rs10796040
45	chr10	44	0.36	0.54	2	0	2	0	NONE	rs12413578, rs144536148, rs186856025
47	chr11	59	0.47	0.53	2	4	2	5	NONE	rs12574838, rs331463
51	chr11	17	0.12	0.36	2	2	8	0	NONE	rs4936059, rs11221402, rs7106876
54	chr12	22	0.22	0.51	2	0	1	1	T CELL, B CELL	rs10774624, rs3184504, rs7310615
56	chr14	12	0.13	0.37	2	3	2	0	ALL	rs1950897, rs911263, rs1885013, rs2104047, rs3784099
57	chr14	21	0.22	0.45	2	7	10	6	ALL	rs7146217, rs36045050, rs11158764
58	chr15	17	0.25	0.48	2	1	12	2	T CELL, B CELL	rs8032939, rs8043085, rs4924273
61	chr16	18	0.08	0.23	2	0	0	0	NONE	rs11075010, rs1579258, rs4584833
63	chr17	39	0.49	0.67	2	0	0	6	NONE	rs7224929, rs58483057, rs2071456
67	chr20	11	0.32	0.59	2	1	1	1	ALL	rs4239702, rs4810485, rs1883832
71	chr21	37	0.32	0.6	2	0	26	0	T CELL	rs1893592, rs225433, rs11203203
72	chr22	103	0.24	0.26	2	9	20	22	T CELL, B CELL	rs11089637, rs11089620, rs5754387

By mapping the credible SNP sets to the annotated active promoters and enhancers in T cells, B cells, and FLS, we further refined nine of 19 category 1 loci to ≤ 3 credible SNPs in active enhancers in either immune cells (*n* = 5 signals), FLS (*n* = 1 signal), or both (*n* = 3 signals) (Table 1, Additional file 8: Table S5 columns L, M, N). Similarly, we narrowed down the number of putative causal SNPs to ≤ 3 for 18 of the 26 category 2 signals, after mapping enhancer marks to the credible set SNPs in immune cells (*n* = 7 signals), FLS (*n* = 3 signals), or both (*n* = 8 signals) (Table 1, Additional file 8: Table S5 columns L, M, N). Thus, by integrating genetic fine mapping with functional chromatin annotation in immune cells and FLS, we identified 27 association signals (37%) that harbor ≤ 3 putative causal RA risk variants having high posterior probabilities and mapping to cell type-specific active enhancers. Examples of the functional genome organization at category signals 1–3 in FLS are shown in Additional file 5: Fig S3-S5.

### Integrative analysis of genetic, expression, and epigenetic datasets links putative causal genes and cell types

We then used our genetic fine mapping and epigenetic datasets to determine candidate effector genes (proximal and interacting in FLS/immune cell types) and their expression in relevant cell types.

In total, 9 of the 73 signals were assigned exclusively to FLS, with 2 further signals assigned to FLS and B cells, and 12 to all three analyzed cell types based on SNPs in cell-type-specific enhancers (Table 2, Additional file 8: Table S5 column based on O, P, Q, labelled in column R). To assign putative target genes to the association signals in FLS, we identified significant CHiC interactions between the regions containing a credible SNP set (CHiC baits, see details in “Methods”) and a gene promoter. We defined gene promoters by downloading all transcripts from Ensembl (version 98) and assigning a 1000-base pair window directly upstream of each transcript as a promoter. In total, we determined 220,000 promoters for 57,602 genes, including noncoding RNA. Across the 73 signals of association, gene target assignments yielded a total of 228 and 227 interacting, expressed FLS target genes in basal and TNF-stimulated conditions, respectively, with 188 gene targets shared between the conditions (Additional file 8: Table S5 columns W and X).

**Table 2:** Genes assigned to FLS based on posterior probability of SNPs in enhancers

Locus	Chr	SNP cate gory	FLS, T, B, ALL, NONE (based on posteriors of SNPs in enhancers)	Top posterior rs numbers	Candidate genes (proximal)	Candidate genes (interacting)	FLS basal proximal genes	FLS basal distal genes	FLS stim. proximal genes	FLS stim. distal genes
3	chr1	2	FLS	rs28411352, rs28489009, rs2306627	POU3F1; MANEAL	RSPO1; RHBDL2	INPP5B; YRDC; C1orf122; MTF1; SF3A3; FHL3	MTF1; GNL2; RRAGC; MYCBP; YRDC; C1orf122; SF3A3	YRDC; C1orf122; MTF1; SF3A3	MTF1; CDCA8; RRAGC; MYCBP; YRDC; C1orf122
4	chr1	1	FLS	rs4839319, rs4839318, rs77227025	PTPN22	RLIMP2; SYT6; BCL2L15	MAGI3; RSBN1; AP4B1; PHTF1 AP4B1-AS1; HIPK1; OLFML3	HIPK1; AP4B1- AS1; OLFML3	MAGI3; AP4B1- AS1; HIPK1; OLFML3	HIPK1; AP4B1- AS1; OLFML3
10	chr2	1	ALL	rs10175798, rs10173253, rs906868, rs7579944, rs1355208			LBH	LCLAT1	LBH	LCLAT1
11	chr2	2	ALL	rs34695944, rs56095903			REL		REL	REL; PUS10
12	chr2	3	FLS	rs1858037, rs1858036, rs11673987, rs11126035, rs906577			SPRED2	ACTR2; SPRED2	SPRED2	ACTR2; SPRED2; AFTPH; PPP3R1
20	chr3	1	ALL	rs73081554, rs185407974, rs180977001, rs35677470, rs114584537	DNASE1L3; HTD2	ALCOX2	FLNB; FLNB-AS1; ABHD6; RPP14; PXK	PXK; SLMAP; FLNB; FLNB-AS1; PDHB	FLNB; FLNB-AS1; PXK	PXK; FLNB; FLNB-AS1; PDHB; SLMAP
23	chr4	3	FLS	rs34046593, rs36020664, rs11933540, rs6448434, rs6448432		SMIM20; TBC1D19; STIM2		RBPJ		RBPJ
25	chr5	3	FLS	rs2561477		PDZPHIP	C5orf30			
27	chr6	3	FLS	rs2234067, rs1885205, rs916287, rs4713969, rs879036				STK38; SRSF3		STK38; SRSF3; MAPK14
28	chr6	3	ALL		TCTE1	SPATS1; VEGFA	AARS2; NFKBIE	HSP90AB1; SLC35B2	NFKBIE	HSP90AB1; SLC35B2; NFKBIE
33	chr7	2	ALL	rs186735625, rs57585717, rs2158624		HOXA11; HOTAIRM1	JAZF1	JAZF1; HOXA10; HOXA11-AS; CREB5	JAZF1	JAZF1; HOXA11- AS; CREB5
34	chr7	3	FLS, B CELL	rs4272, rs8179, rs42034		SAND9; HEPACAM2; VPS50	CDK6	CDK6; SAMD9; SAMD9L	CDK6	CDK6; SAMD9; SAMD9L
36	chr8	2	FLS, B CELL	rs2736337	BLK	TDH; GATA4; DEFB				
38	chr8	2	FLS	rs678347, rs507201, rs657425	GRHL2			RRM2B		
41	chr9	3	ALL	rs10985070			C5; PHF19; TRAF1	PHF19; MEGF9; CNTRL; FBXW2; PSMD5; GSN; GSN-AS1	PHF19; TRAF1	PHF19; TRAF1; CNTRL; FBXW2; GSN; GSN-AS1; PSMD5; STOM
46	chr10	1	ALL	rs12764378, rs71508903		RTKN2	ARID5B	ARID5B	ARID5B	ARID5B
48	chr11	3	ALL	rs968567, rs7943728, rs61896141, rs61897793, rs61897795	CD5	PTGDR2	FADS2; SLC15A3; TKFC; INCENP; CCDC86; PRPF19; TMEM109; TMEM132A; VPS37C; DDB1; CYB561A3; TMEM138; CPSF7; MYRF; FEN1; FADS1; FADS3; BEST1; FTH1	FADS2; FADS1; MYRF; FEN1; INCENP; FADS3; BEST1; FTH1; RAB3IL1; CCDC86; PRPF19; TMEM109	FADS2; SLC15A3; TKFC; INCENP; CCDC86; PRPF19; TMEM109; TMEM132A; VPS37C; DDB1; CYB561A3; TMEM138; CPSF7; MYRF; FEN1; FADS1; FADS3; BEST1; FTH1	FADS2; FADS1; MYRF; FEN1; VPS37C; INCENP; FADS3; BEST1; FTH1; CCDC86; PRPF19; TMEM109; TMEM132A; SLC15A3
56	chr14	2	ALL	rs1950897, rs911263, rs1885013, rs2104047, rs3784099			RAD51B	ZFP36L1		ZFP36L1
57	chr14	2	ALL	rs7146217, rs36045050, rs11158764			RAD51B;ZFYVE26 ; ZFP36L1	ZFP36L1	ZFYVE26; ZFP36L1	ZFP36L1
67	chr20	2	ALL	rs4239702, rs4810485, rs1883832			CD40	SLC35C2; ELMO2		ELMO2; SLC35C2; NCOA5
68	chr21	3	ALL	rs73194058, rs11702844, rs11700997			SON; IFNGR2; ITSN1; TMEM50B; GART; DONSON; CRYZL1	IFNGR2; GART; SON; ITSN1; IFNAR1; PAXBP1- AS1; IL10RB-DT; DONSON; NDUFV3; ATP5PO	SON; IFNGR2; ITSN1; GART; DONSON	IFNGR2; GART; SON; ITSN1; IFNAR2; IFNAR1; PAXBP1; IL10RB- DT; IL10RB; DONSON; PTER
69	chr21	3	FLS	rs7278771, rs7283600, rs147868091			RCAN1	RCAN1	RCAN1	RCAN1
70	chr21	1	FLS	rs8133843, rs8129030, rs9979383		SET4; PPP1R2P2		RUNX1		RUNX1; CLIC6

Credible SNPs in category 1 and 2 signals found predominantly in FLS implicated genes including *GRHL2*, *MYCBP*, and *RUNX1* (Table 2, Additional file 5: Figure S3). FLS-assigned genes that were associated with category 3 association signals, which showed negligible posterior probability (< 2%) in immune enhancer SNPs, included *SPRED2*, *RCAN1*, *CDK6*, and *RBPJ* (Table 2, Additional file 5: Figure S5). Notably, the 24 credible SNPs in the *RBPJ* association signal and the 41 credible SNPs in the *CDK6* association signal were reduced to just six and three SNPs, respectively, mapping to FLS-specific enhancers. The *RBPJ* SNPs were localized in FLS-specific enhancers, with none found in T or B cells (Additional file 8: Table S5, rs11933540). This indicated that the putative causal SNPs in the *RBPJ* association signal might specifically affect the function of FLS in RA.

We then integrated the credible set SNPs with our previously established CHiC dataset from B cell (GM12878) and T cell (Jurkat) lines [12, 19]. We found that the RA credible sets assigned to immune cell types associated with genes that are vital in T and B cell-specific activities (Table 3, Additional file 8: Table S5 columns AA to AD). Genes in category 1 and 2 signals, which associated with active immune cells enhancer regions, included *CTLA4*, *IL2RA*, and *GATA3* for T cells and *BLK* for B cells (Table 3, Additional file 8: Table S5 columns AA to AD). Of note, the *ANKRD55/IL6ST* locus (rs7731626 in Additional file 8: Table S5) had a single SNP in the credible set, an eQTL with both *ANKRD55* and *IL6ST* [20] confined to an enhancer exclusive to T cells in our analysis. Immune cell-assigned genes from category 3, where credible SNPs in immune enhancers accounted for > 30% of the posterior probability, but had negligible posterior probability (< 5%) in FLS enhancers, included *STAT4*, *CXCR5*, *CD28*, and *MYC*.

**Table 3:** Genes assigned to T and/or B cells based on posterior probability of SNPs in enhancers

Locus	Chr	SNP cate gory	FLS, T, B, ALL, NONE (based on posteriors of SNPs in enhancers)	Top posterior rs numbers	Jurkat T cells proximal genes	Jurkat T cells distal genes	GM12878 B cells proximal genes	GM12878 B cells distal genes
5	chr1	1	B CELL	rs6679677, rs2476601	RSBN1; PHTF1; PTPN22	RSBN1; PHTF1; PTPN22; AP4B1; HIPK1-AS1; HIPK1; OLFML3	RSBN1; PHTF1; PTPN22	RSBN1; PTPN22; PHTF1; AP4B1-AS1; BCL2L15; AP4B1; HIPK1-AS1; HIPK1; OLFML3; LRIG2
6	chr1	2	B CELL	rs1217404, rs2476604, rs1217420	RSBN1; PHTF1; PTPN22; AP4B1-AS1	RSBN1; PHTF1; PTPN22; AP4B1; HIPK1-AS1; HIPK1; OLFML3	RSBN1; PHTF1; PTPN22; AP4B1-AS1	PTPN22; RSBN1; PHTF1; AP4B1-AS1; BCL2L15; AP4B1; HIPK1-AS1; HIPK1; OLFML3; LRIG2; FRMD8; ARL13B; STX19
8	1	3	B CELL	rs4657041, rs1801274, rs6671847	FCGR2A	FCER1G; NDUFS2; SDHC; MPZ; CFAP126; FCGR2A; FCGR2B; FCRLA; FCRLB; RN7SL466P; DUSP12; ATF6; PCP4L1; ADAMTS4	FCGR2A	SDHC; MPZ; FCGR2A; FCGR2B; FCRLA; FCRLB; RN7SL466P; DUSP12; ATF6; CFAP126; RNU6- 481P; ADAMTS4; NDUFS2
10	chr2	1	ALL	rs10175798, rs10173253, rs906868, rs7579944	LBH	LBH	LBH	LBH; LCLAT1
11	chr2	2	ALL	rs34695944, rs56095903, rs67574266, rs13031237, rs13031721	LINC01185; REL; RNU4- 51P	LINC01185; REL; RNU4- 51P; PUS10; PAPOLG; RN7SL632P; RNA5SP95; B3GNT2; RNU6-612P	LINC01185; REL; RNU4- 51P	LINC01185; REL; RNU4- 51P; PUS10; PAPOLG; RN7SL632P; RNA5SP95; B3GNT2; RNU6-612P
14	chr2	3	T CELL	rs13426947, rs3024859, rs7568275, rs11889341	STAT4	STAT4; RNU6-959P; MYO1B	STAT4	STAT4; RNU6-959P
16	chr2	3	T CELL	rs1980421, rs1980422, rs7588874, rs7422494	CD28; RNU6-474P; CTLA4	CD28; RNU6-474P; CTLA4; RAPH1; ABI2	CD28; RNU6-474P; CTLA4	CD28; CTLA4; RNU6-474P; RAPH1; PRKG1
17	chr2	1	T CELL	rs231724, rs231723, rs231775	CTLA4	CTLA4; CD28; RNU6- 474P; RAPH1	CTLA4	CD28; CTLA4; RNU6- 474P; RAPH1
19		2	T CELL, B CELL	rs9310852, rs4680838, rs9880772, rs1353286	EOMES	LINC02084; EOMES; LINC01967; CMC1; AZI2; ZCWPW2; NEK10; LINC01980	EOMES	LINC02084; EOMES; CMC1; AZI2; ZCWPW2
20	chr3	1	ALL	rs73081554, rs185407974, rs180977001, rs35677470, rs114584537	FLNB; DNASE1L3; FLNB- AS1; ABHD6; RPP14; HTD2; PXK	DNASE1L3; ABHD6; RPP14; HTD2; PXK; PDHB; KCTD6; ACOX2; FAM107A; FAM3D-AS1; FAM3D; FLNB	FLNB; DNASE1L3; FLNB- AS1; ABHD6; RPP14; HTD2; PXK	DNASE1L3; ABHD6; RPP14; HTD2; PXK; FLNB; FLNB-AS1; PDHB; KCTD6; ACOX2; FAM107A; FAM3D-AS1; FAM3D
24	chr5	1	T CELL	rs7731626	ANKRD55	ANKRD55; RNA5SP184; IL6ST	ANKRD55	ANKRD55; RNU6-299P; RNA5SP184; IL31RA; IL6ST
28	chr6	3	ALL		TMEM151B; AARS2; NFKBIE; TCTE1	TMEM151B; AARS2; TCTE1; HSP90AB1; SLC35B2; MIR4647; NFKBIE; SPATS1; CAPN11; TMEM63B; RN7SL811P	TMEM151B; AARS2; NFKBIE; TCTE1	TMEM151B; AARS2; TCTE1; NFKBIE; HSP90AB1; SLC35B2; MIR4647; MRPL14; TMEM63B; SPATS1; TRIM38; CAPN11; MTX2
31	chr6	2	B CELL	rs2451258, rs2485363, rs654690 , rs1994564, rs212389		RSPH3; TAGAP; SYTL3; C6orf99		RSPH3; TAGAP; SYTL3; C11orf44
32	chr6	1	T CELL, B CELL	rs1571878, rs3093017, rs10946216	CCR6	CCR6; RPS6KA2	CCR6	CCR6; SFT2D1; RPS6KA2; RNASET2; MIR3939; FGFR1OP; GPR31; PPIL4
33	chr7	2	ALL	rs186735625, rs57585717, rs2158624	JAZF1; JAZF1-AS1; RNU6- 979P	JAZF1; RNU6-979P; JAZF1-AS1; HOTTIP; HOXA1; HOTAIRM1; HOXA3; HOXA-AS2; HOXA4; HOXA5; HOXA6; HOXA-AS3; HOXA7; HOXA9; HOXA10-AS; MIR196B; HOXA10; HOXA11; HOXA11-AS; EVX1-AS; EVX1	JAZF1; JAZF1-AS1; RNU6- 979P	JAZF1; RNU6-979P; JAZF1-AS1; HOTTIP; HOXA11; HOXA11-AS; HOXA1; HOTAIRM1; HOXA3; HOXA-AS2; HOXA4; HOXA5; HOXA- AS3; HOXA7; HOXA9; HOXA10-AS; MIR196B; HOXA10; EVX1-AS; EVX1; HOXA6; PARP9
34	chr7	3	FLS, B CELL	rs4272, rs8179, rs42034	CDK6	CDK6; PEX1; RBM48; FAM133B; CDK6-AS1; SAMD9; VPS50; HEPACAM2	CDK6	CDK6; FAM133B; SAMD9; CDK6-AS1

These analyses highlighted a number of SNP-enhancer-gene combinations that could be assigned to an immune cell or fibroblast-driven risk of developing RA. We were able to assign > 60% of the non-HLA RA association signals with a putative causal cell type (FLS, B cells, T cells) and putative causal gene (Additional file 8: Table S5 column R not “none”). Compared to previous gene assignment results [2], our method provides empirical evidence for an additional 104 RA-associated genes at the 73 European association signals.

### TNF-induced alterations in 3D chromatin structure assign additional RA risk genes to FLS

At 17 of the 73 associated signals, we observed a change in chromatin interactions in stimulated FLS, which were linked to 35 genes (Table 4). RNA-seq showed that the expression of 17 of the 35 genes was increased upon TNF stimulation in FLS (FDR < 0.05) (e.g., *TRAF1*, *TNFAIP3*, *IFNAR2)* (Table 4, Additional file 5: Fig S6). Nine of the 35 genes were downregulated after TNF in FLS (FDR < 0.05) (e.g., *RBPJ* and *RNF41*) (Table 4).

**Table 4:** List of all loci with significantly changed chromatin interactions after TNF stimulation and expression of associated genes after TNF stimulation

Locus	Interacting gene	Base mean expression	log2Fold Change	lfcSE	stat	***p*** value	padj
12	SPRED2	772.22	− 0.34	0.08	− 4.36	1.29E−05	7.23E−05
19	SLC4A7	3702.62	− 0.23	0.13	− 1.80	0.072	0.145
20	PXK	1012.38	− 0.33	0.11	− 3.05	0.002	0.008
**23**	**RBPJ**	**5319.00**	− **0.56**	**0.22**	− **2.51**	**0.012**	**0.032**
**24**	**IL6ST**	**18,850.31**	**0.72**	**0.08**	**8.91**	**4.91E**−**19**	**2.30E**−**17**
**30**	**TNFAIP3**	**7841.42**	**4.57**	**0.19**	**23.43**	**2.14E**−**121**	**3.51E**−**118**
**34**	**CDK6**	**4906.40**	**0.92**	**0.12**	**7.89**	**3.05E**−**15**	**9.21E**−**14**
35	TNPO3	1671.71	0.05	0.07	0.76	0.449	0.597
**41**	**PHF19**	**1065.75**	**0.71**	**0.14**	**5.09**	**3.56E**−**07**	**2.74E**−**06**
**42**	**TRAF1**	**1041.51**	**2.80**	**0.22**	**12.65**	**1.14E**−**36**	**1.98E**−**34**
**48**	**VPS37C**	**552.68**	**0.71**	**0.07**	**10.56**	**4.76E**−**26**	**3.90E**−**24**
**52**	**CDK2**	**648.74**	**0.85**	**0.13**	**6.69**	**2.23E**−**11**	**3.72E**−**10**
52	RAB5B	1805.92	− 0.24	0.06	− 4.06	4.88E−05	2.42E−04
**52**	**RNF41**	**1878.98**	− **0.63**	**0.11**	− **5.62**	**1.93E**−**08**	**1.88E**−**07**
52	ANKRD52	1954.38	0.23	0.07	3.09	0.002	0.007
52	CNPY2	1033.14	0.37	0.07	5.30	1.16E−07	9.89E−07
52	PAN2	312.46	− 0.23	0.08	− 2.80	0.005	0.015
**52**	**STAT2**	**4951.27**	**0.74**	**0.20**	**3.77**	**1.64E**−**04**	**7.21E**−**04**
**52**	**TIMELESS**	**728.81**	**1.11**	**0.14**	**8.10**	**5.66E**−**16**	**1.87E**−**14**
52	GLS2	18.80	0.34	0.26	1.31	0.191	0.317
53	SLC26A10	10.97	− 0.29	0.39	− 0.75	0.455	0.603
53	OS9	5461.71	− 0.12	0.07	− 1.83	0.068	0.138
53	AGAP2	5.83	0.43	0.31	1.36	0.173	0.294
53	TSPAN31	370.51	− 0.29	0.11	− 2.56	0.010	0.028
53	CDK4	1630.73	0.06	0.07	0.88	0.378	0.529
53	AVIL	64.47	− 0.44	0.15	− 2.93	0.003	0.011
53	CTDSP2	3707.75	− 0.47	0.06	− 7.79	6.45E−15	1.85E−13
**54**	**SH2B3**	**1493.15**	**0.52**	**0.10**	**5.29**	**1.25E**−**07**	**1.06E**−**06**
54	ATXN2	781.23	− 0.05	0.08	− 0.71	0.478	0.625
64	ORMDL3	800.27	0.40	0.08	4.75	1.99E−06	1.32E−05
64	PSMD3	1842.20	0.18	0.05	3.66	2.53E−04	0.001
**65;68**	**IFNAR2**	**644.38**	**2.81**	**0.11**	**26.14**	**1.14E**−**150**	**2.92E**−**147**
**68**	**IFNAR1**	**2355.18**	**0.95**	**0.08**	**12.55**	**3.95E**−**36**	**6.53E**−**34**
**68**	**IFNGR2**	**1248.98**	**1.10**	**0.08**	**12.90**	**4.71E**−**38**	**9.03E**−**36**
68	ITSN1	1138.10	− 0.24	0.11	− 2.10	0.036	0.080

Genes with a log2fold change > ± 0.5 and padj < 0.05 are marked in bold. *lfcSE* standard error for log2 fold change, *stat* statistic value for the null hypothesis, *padj p* value adjusted for multiple testing using Benjamini-Hochberg

Since TADs have been shown to define probable limits of gene regulation, we also overlapped TADs with the association signals. We observed that each credible SNP set is usually found within one or two adjacent TADs. We then examined the genes within these TADs in basal and stimulated FLS and found that alterations in TAD boundaries after stimulation led to different genes being overlapped by associated TADs. Genes found within stimulation-specific TADs included *TNFAIP3, JAZF1, ZFP36L1, INFGR1,* and *LBH.* Several of these genes, including *TNFAIP3, JAZF1, IFNGR1,* and *LBH,* also showed differential gene expression between basal and stimulated states (Table 5, Additional file 8: Table S5 columns AH and AI).

**Table 5:** Alterations in TAD structure after TNF stimulation and genes in stimulation-specific TADs

Locus	Chr	TAD_diff	Type of difference	Enriched_in	Expressed in FLS	Differential expressed in FLS (***p*** adj < 0.05)
9	chr1	Differential	Strength Change	Basal	RC3H1; RABGAP1L; PRDX6; KLHL20; GAS5-AS1; GAS5; ZBTB37	GAS5; PRDX6; ZBTB37; RABGAP1L; RC3H1
10	chr2	Differential	Complex	Basal	LBH; LCLAT1; YPEL5	LBH; LCLAT1
13	chr2	Differential	Merge	Stim	NPAS2	NPAS2
19	chr3	Differential	Strength Change	Basal	CMC1; SLC4A7; AZI2	CMC1
20	chr3	Differential	Split	Basal	SLMAP; FLNB; FLNB-AS1; PXK; PDHB	PXK; FLNB
21	chr3	Differential	Strength Change	Basal	PCCB; PPP2R3A; MSL2; STAG1	PCCB
22	chr4	Differential	Strength Change	Basal	WDR1; ZNF518B	
27	chr6	Differential	Strength Change	Basal	SRPK1; MAPK14; PI16; LHFPL5; BRPF3; KCTD20; STK38; SRSF3; PANDAR; CDKN1A; C6orf89; MTCH1	SRPK1; C6orf89; STK38; MTCH1; KCTD20; BRPF3; CDKN1A; SRSF3
28	chr6	Differential	Strength Change	Stim	RSPH9; VEGFA; CDC5L; TMEM63B; HSP90AB1; SLC35B2	TMEM63B; CDC5L
29	chr6	Differential	Strength Change	Stim	IFNGR1; WAKMAR2; TNFAIP3	TNFAIP3; IFNGR1
30	chr6	Differential	Strength Change	Stim	IFNGR1; WAKMAR2; TNFAIP3	TNFAIP3; IFNGR1
32	chr6	Differential	Strength Change	Basal	RPS6KA2; AFDN	
33	chr7	Differential	Strength Change	Stim	CREB5; TAX1BP1; JAZF1	JAZF1; TAX1BP1
47	chr11	Differential	Strength Change	Basal	TRIM44; FJX1; COMMD9	COMMD9
48	chr11	Differential	Strength Change	Basal	FADS2; SLC15A3; TKFC; INCENP; AHNAK; INTS5; CCDC86; PRPF19; TMEM109; TMEM132A; VPS37C; DDB1; CYB561A3; TMEM138; CPSF7; MYRF; FEN1; FADS1; FADS3; RAB3IL1; BEST1; FTH1; EEF1G; TUT1; MTA2; EML3; ROM1; GANAB; LBHD1; CSKMT; UQCC3; UBXN1; LRRN4CL; HNRNPUL2-BSCL2; HNRNPUL2; TMEM179B; TMEM223; NXF1; STX5; RNU2-2P; SLC3A2	TMEM132A; FTH1; SLC15A3; FADS1; AHNAK; VPS37C; INCENP; RAB3IL1; LRRN4CL; EEF1G; TMEM138; ROM1; TUT1; DDB1; FADS2; CCDC86; MTA2; TMEM109
49	chr11	Differential	Strength Change	Basal	ACAT1; ATM; CUL5; NPAT; POGLUT3	ATM; ACAT1; NPAT; CUL5
57	chr14	Differential	Strength Change	Basal	ZFYVE26; ZFP36L1	
65	chr18	Differential	Split	Basal	SPIRE1; SEH1L; CEP192; AFG3L2; CEP76	CEP76; CEP192; SPIRE1; SEH1L
72	chr22	Differential	Strength Change	Basal	MAPK1; UBE2L3; PPIL2; YPEL1; PPM1F	MAPK1; UBE2L3
73	chr22	Differential	Strength Change	Basal	JOSD1; GTPBP1; SUN2; CBX6; APOBEC3C; CBX7; RPL3; MIEF1; ATF4; RPS19BP1	GTPBP1; RPL3; SUN2; ATF4; CBX6

The correlated change in chromatin structure, interaction strength of RA implicated regions, and gene expression upon stimulation demonstrated how these loci are dynamic and active in FLS and suggests that RA-associated variants could affect the transcriptional response to TNF in FLS.

### TNF stimulation induces major changes in chromatin organization of the TNFAIP3 and IFNGR2 association signals with concomitant effects in the expression of interacting genes in FLS

Some of the RA association signals emerged as particularly interesting in FLS, exemplifying how stimulation-induced changes in chromatin conformation and gene expression can affect RA risk in FLS.

**Fig. 5:**
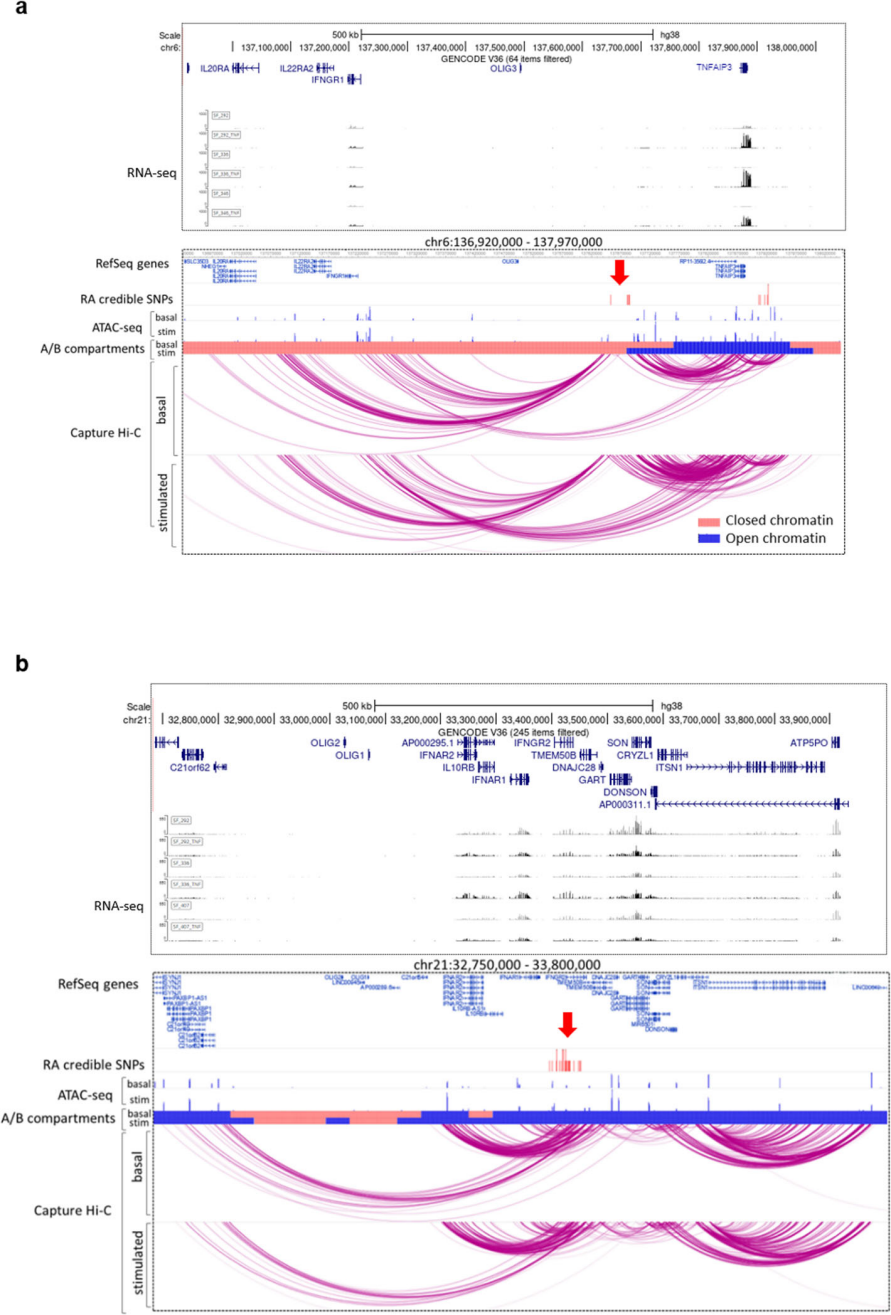
TNFAIP3 and IFNAR1 genetic risk loci—exemplary regions linked to TNF stimulation. Two exemplary risk regions where TNF stimulation had profound effects on chromatin structure and influenced the genetic regions containing RA SNPs. **a** The TNFAIP3 region on chromosome 6q23 (red arrow) containing RA credible SNPs (red lines, rs17264332 in Additional file 8: Table S5) changed from closed chromatin (light red bar) to open chromatin state (blue bar) after TNF stimulation and exhibited increased interactions with the promoter of *TNFAIP3* in stimulated FLS (see also Table 4). **b** The genomic IFNGR2 region of the credible SNP set on chromosome 21 (rs73194058 in Additional file 8: Table S5, red arrow) interacted with several nearby genes. These interactions were further enhanced by TNF stimulation (see also Table 4). Chromatin at the IFNAR2 gene locus changed from a closed (light red bar) to open (blue bar) state in stimulated FLS. RNA-seq tracks show one randomly chosen RA hand, RA knee, and RA shoulder sample with (black) or without (gray) TNF stimulation

The intergenic region on chromosome 6q23 between *OLIG3* and *TNFAIP3*, which contains eight credible SNPs (rs17264332 in Additional file 8: Table S5), was dynamically linked to the *TNFAIP3* gene through DNA accessibility, chromatin interactions, and gene expression. The organization of this genomic region changed from a closed, inactive (compartment B) to an open, active chromatin conformation (compartment A) upon TNF stimulation of FLS (Fig. 5a), and TAD boundary strength increased in TNF-stimulated FLS (Table 5). These substantial alterations to the chromatin organization coincided with a strong increase in the expression of the interacting *TNFAIP3* gene in FLS (Fig. 5a, Table 4, Additional file 5: Fig S6).

Similarly, we demonstrated stimulation-induced changes in chromatin activity in the *IFNGR2* region (rs73194058, Additional file 8: Table S5) in FLS. Our CHiC analysis showed that the credible set SNPs in this region interacted with several genes relevant to the interferon (IFN) pathway, such as *IFNAR2*, *IL10RB, IFNAR1,* and *IFNGR2* (Additional file 8: Table S5 columns W to Z and Fig. 5b). TNF stimulation of FLS induced dynamic changes in chromatin interactions at this locus and increased the expression of *IFNAR2, IFNAR1*, and *IFNGR2* (Additional file 5: Fig S1, Table 4, Fig. 5b). Additionally, chromatin accessibility in the region of *IFNAR2* changed from an inactive B to an active A compartment in stimulated FLS (Fig. 5b).

The *TNFAIP3/IFNGR1* region on chromosome 6 and the *IFNAR1/IFNGR2* region on chromosome 21 interacted with genes encoding five subunits of the IFN I/III receptors in FLS (Fig. 5a,b), suggesting a close genetic link between FLS function and IFN response in RA.

### Genes linked to RA risk SNPs in FLS are functionally interlinked and regulate FLS-relevant RA functions

To predict biological processes influenced by potential transcriptional effects of risk variants active in FLS, we conducted analyses to predict protein-protein interaction, pathway enrichment, and functional annotation clustering. For these analyses, we included all target genes of the RA association signals that were assigned to FLS as a causal cell type (“All” and/or “FLS” in column R of Additional file 8: Table S5).

**Fig. 6:**
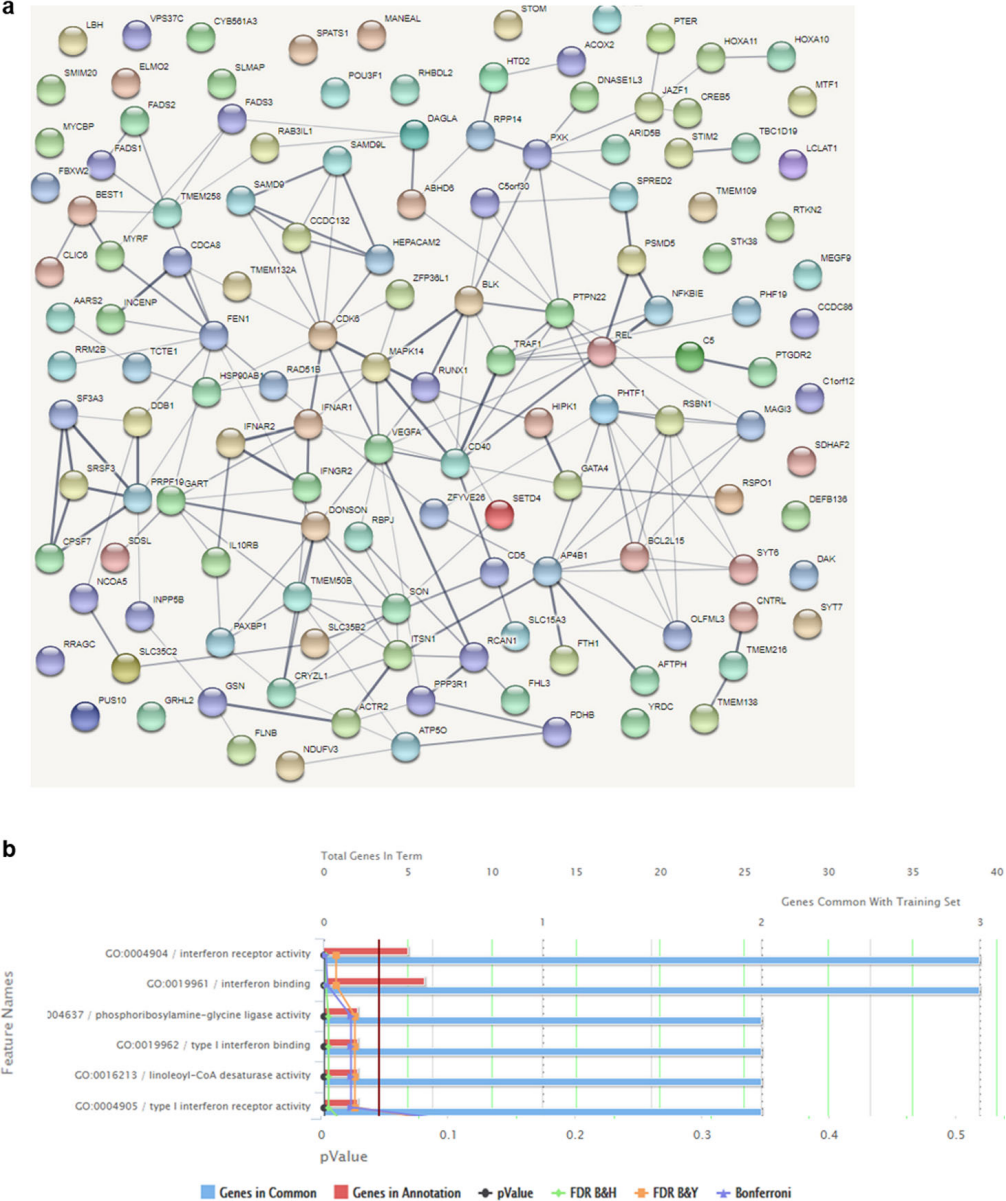
Predicted functional networks of genes that were associated with SNPs active in FLS. **a** A protein-protein interaction network was established using STRING with default settings (medium confidence). The obtained network had more interactions than expected by chance with a protein-protein-interaction enrichment *p* value of 1.28e−08. The thickness of the lines indicates the strength of data support. Colors, distances, and location on the map were assigned randomly. **b** Functional enrichment of genes interacting with SNPs active in FLS was detected using ToppFun in default settings. Significant terms for GO molecular function are shown. FDR = false discovery rate; B&H = Benjamini-Hochberg ; B&Y = Benjamini-Yekutieli

We found significantly enriched protein-protein interactions for the genes in the loci active in FLS by using STRING protein-protein interaction networks (PPI enrichment *p* value < 1.0e−016; Fig. 6a) and identified additional functional connections between the assigned genes by literature search. For instance, *ZFP36L*, *CDK6,* and *RUNX1* were all assigned to signals active in RA (Additional file 8: Table S5 column R), are functionally connected, and regulate cell proliferation. *CD40* (rs4239702 in Additional file 8: Table S5), *RBPJ* (rs11933540 in Additional file 8: Table S5), and *TRAF1* (rs10985070 in Additional file 8: Table S5) may constitute another genetically influenced interlinked functional network in FLS.

Gene Ontology (GO) molecular function analysis (Fig. 6b) and functional annotation clustering of enriched pathways with the genes associated with credible set SNPs in FLS (Additional file 8: Table S5 column R and columns U-Z) revealed several clusters highly relevant to RA pathogenesis. These clusters included enrichment of genes involved in IFN response and viral defence (*IFNAR1, IFNAR2, CD40, IFNGR, C5, IL-10RB*) (Database for Annotation, Visualization and Integrated Discovery DAVID [21, 22] enrichment score 1.22), as well as lipid metabolism and fatty acid synthesis (*FADS1-3, ACOX2, LCLAT1, JAZF1, DAGLA*) (DAVID enrichment score 1.91). In addition, “cilium morphogenesis” emerged as an enriched term (DAVID enrichment score 0.87) and several genes associated with RA risk SNPs in FLS were connected to the formation of the primary cilium (*C5orf30, GSN, TMEM138, TMEM216, CNTRL, INCENP, ACTR2*).

Overall, by integrating epigenetic and transcriptional data in FLS, we identified several functional relationships among RA risk variants and their target genes active in FLS. The multi-level effects of RA risk variants on key signalling pathways may contribute to the accumulated genetic risk in driving FLS activation and proliferation in RA.

### RA risk SNPs in the *RBPJ* enhancer region confer joint-specific genetic effects in FLS

**Fig. 7:**
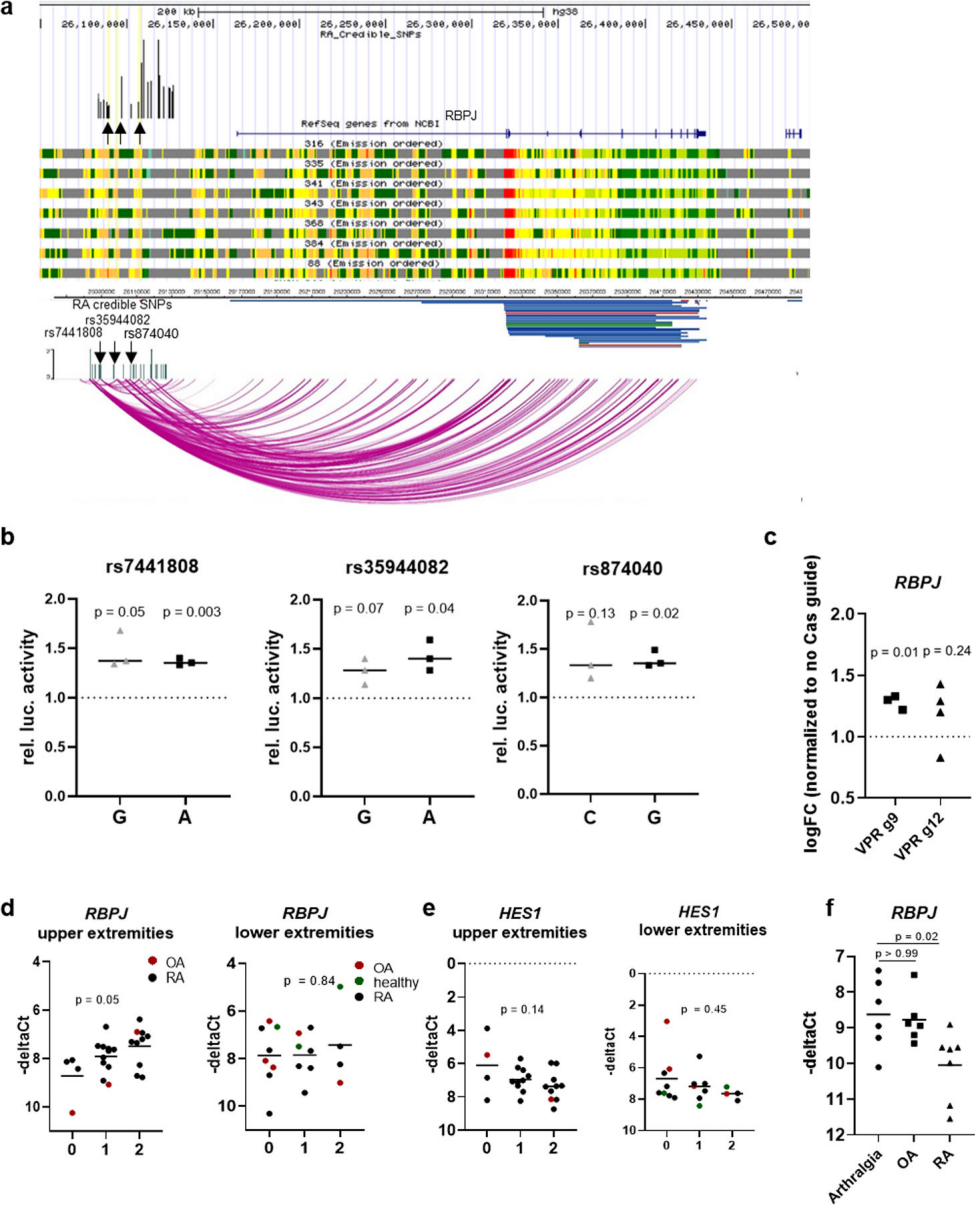
*RBPJ* expression in FLS is affected by genotype and disease. **a** Fine mapping, epigenetic and chromatin conformation analyses at the *RBPJ* locus. Black arrows indicate rs7441808, rs35944082, and rs874040, which were selected for further analysis. **b** Luciferase reporter assays showing relative enhancer activity of oligonucleotides containing risk (gray) and wild-type variants (black) of rs7441808, rs35944082, and rs874040 compared to empty vectors (set to 1). One sample *t* test. **c** *RBPJ* expression in FLS transduced with VP64-p65-Rta dCas9 (VPR) and two different guide RNAs (g9 and g12) targeting the genomic region around chr4:26106575 (rs87040). *RBPJ* expression was normalized to FLS that were transduced with respective guide RNAs but not VPR-dCas9 (set to 1). –deltaCt = cycle of threshold of *RBPJ* expression—cycle of threshold *RPLP0*. One sample *t* test. **d** *RBPJ* expression in FLS isolated from individuals homozygous for rs874040 in the locus near the *RBPJ* gene (0), heterozygous (1), or homozygous for the wild-type variant (2). Upper extremity joints included joints of the hand, elbows, and shoulders; lower extremity joints included hips, knees, and joints of the feet. One-way ANOVA. **e** Expression of *HES1* in the same FLS cohort. One-way ANOVA. **f** *RBPJ* expression in individuals with joint pain, but no histological signs of arthritis (arthralgia), OA and RA. One-way ANOVA with Bonferroni correction

Our epigenetic and functional analyses of the *RBPJ* association signal identified *RBPJ* as a candidate causal and functional gene in FLS (rs11933540 in Additional file 8: Table S5). Mapping of the 24 credible SNPs to FLS enhancers in the *RBPJ* association signal reduced the number of likely causal SNPs to six, which lie in active chromatin in FLS (Additional file 8: Table S5 rs11933540). To confirm enhancer activity of these regions, we selected three SNPs in three different regions within the *RBPJ* association signal (rs7441808, rs35944082, rs874040, Fig. 7a). We cloned oligonucleotides (31 bp) with the respective risk and non-risk variants in the middle into luciferase promoter vectors and transfected them into a human fibroblast cell line (HT1080). All three regions showed enhancer activity; however, luciferase activity was similar for both alleles (Fig. 7b). For rs874040, chromatin conformation analysis showed direct interactions with the *RBPJ* gene (Fig. 7a). To functionally establish that the rs874040-containing enhancer region can regulate the expression of *RBPJ*, we transduced FLS with lentiviral particles containing dCas9-VPR and two guide RNAs (g9 or g12) targeting the rs874040-containing enhancer region (Additional file 5: Fig S7). FLS transduced with the activating dCas9-VPR and guide RNAs increased the expression of *RBPJ* compared to FLS transduced with the respective guide RNAs without dCas9-VPR (Fig. 7c). Even though the upregulation of *RBPJ* expression was modest (30%), which could be due to enhancer redundancy in this region and is consistent with previous data showing the limited efficiency of enhancer activation with the dCas9-VPR system [23], this experiment verified the regulation of *RBPJ* expression by the rs874040-containing enhancer region.

FLS homozygous for the risk allele of rs874040 exhibited lower expression of *RBPJ* mRNA compared to FLS with the wild-type variant. This effect was, however, present only in FLS from upper extremity joints, and not from lower extremity joints (Fig. 7d). It is known that RBPJ binds to the promoter of HES1 and represses its transcription [24]. Accordingly, the expression of *HES1* was increased in FLS from patients homozygous for rs874040 in upper extremity joints (Fig. 7e). TNF stimulation significantly downregulated *RBPJ* mRNA expression in FLS (Table 4), and FLS from RA patients expressed less *RBPJ* than FLS from patients with arthralgia (Fig. 7f). These data indicate that genetic predisposition and a pro-inflammatory environment can affect *RBPJ* expression in FLS, which might lead to increased activation of the Notch signalling pathway via HES1.

**Fig. 8:**
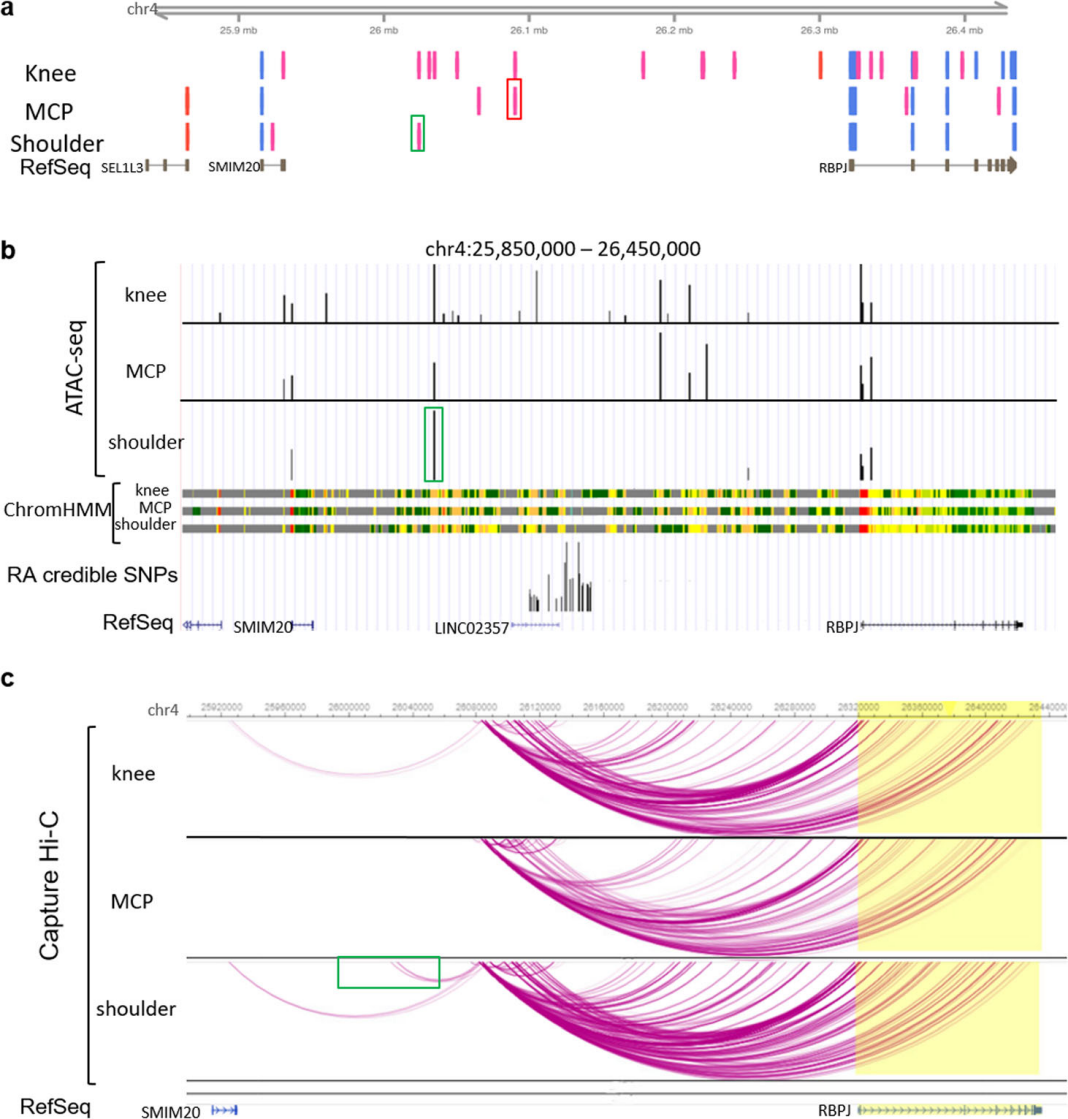
Joint-specific enhancers and chromatin interactions in the *RBPJ* locus might influence the joint-specific expression of *RBPJ*. **a** CAGE measurements of active enhancers (pink bars) and active promoters (light blue bars) in FLS from knees (*n* = 2), metacarpophalangeal (MCP) joints (*n* = 3), and shoulders (*n* = 2). Red box highlights enhancer used in MCP joints overlapping risk SNPs. Green box highlights main enhancer in shoulders. **b** Representative depiction of open chromatin as measured by ATAC-seq (black bars) in FLS from knees (*n* = 3), MCP joints (*n* = 2), and shoulders (*n* = 2) and representative depiction of ChromHMM regulatory regions in FLS from knees (*n* = 3), MCP joints (*n* = 2), and shoulders (*n* = 2). Green box highlights main enhancer in shoulders. **c** Chromatin interactions in FLS from knees (*n* = 2), MCP joints (*n* = 2), and shoulders (*n* = 2) as measured by CHiC. Green box highlights interaction of the shoulder enhancer with the risk locus

To explain the joint-specific effect of rs874040, we explored the enhancer landscape and the chromatin interactions in different upper and lower extremity joints. CAGE-seq data showed that the enhancer activity within the *RBPJ* locus is higher in knee FLS compared to shoulder and hand FLS (Fig. 8a). ATAC-seq peaks largely overlapped with CAGE-seq enhancer signals, being more abundant in knee FLS than in shoulder or hand FLS (Fig. 8b). Shoulder FLS appeared to mainly use an upstream enhancer that interacted with the *RBPJ* risk locus (green boxes, Fig. 8a–c). Overlap of ATAC-seq and CAGE-seq analyses was weaker in hand FLS, but CAGE-seq data indicated that hand FLS used an enhancer within the risk locus (red box, Fig. 8a). Additionally, chromatin interactions within the locus were generally weaker in hand FLS (Fig. 8c). Knee FLS activated several enhancers (Figs. 8a,b) and exhibited strong chromatin interactions across the locus (Fig. 8c). We analyzed DNA-binding motifs in the enhancer overlapping the RA risk locus spanning chr4:26090045-26090465 (hg19) (red box in Fig. 8a) by using the JASPAR2020 database [25]. This enhancer contained TFBS for different HOX transcription factors (HOXA6, HOXA7, HOXA10, HOXB2, HOXB6, HOXB7, HOXB13, HOXD3, HOXD9, HOXD13), similar to the DNA motifs identified in open chromatin at repressed genes after TNF stimulation in FLS (Fig. 3d), which are expressed in a joint-specific manner in FLS [26].

Together, these data suggest that joint-specific differences in chromatin interactions and enhancer usage could underlie the joint-specific effects of rs874040 on *RBPJ* expression in upper extremity joints. This illustrates that RA genetic risk can be different between the joints, thereby shaping a specific pattern of joint involvement in RA.

## Discussion

Deciphering the role of causal genetic variants underlying GWAS loci in RA, albeit challenging, provides an unbiased strategy to understand the core disease pathways and guide drug discovery [2, 27]. Here we demonstrate that a significant proportion of the 73 European ancestry non-HLA RA association signals contain disease-associated variants that are located within active regulatory DNA elements in FLS. Linking these DNA regions with target genes indicates genes and biological pathways that trigger RA susceptibility by stromal cell activation in the joint. Thus, we provide for the first time substantial evidence for an independent, causal role of FLS in RA genetic susceptibility and pathogenesis.

As a first step in our analysis, we created a comprehensive map of the epigenetic landscape of FLS with and without TNF stimulation and assessed key regulators of the transcriptional response of FLS to TNF. By increasing the resolution of our measurements from A/B compartments to TADs and chromatin interactions, we found substantial changes in 3D structure of the FLS genome upon TNF stimulation. We confirmed AP-1, which is intimately linked to the pathogenesis of RA [28], as major transcription factor regulating changes in gene expression of FLS after TNF. AP-1 binding sites were enriched at enhancer sites of genes with increasing as well as repressed gene expression in TNF-stimulated FLS. This is in line with findings that different subunits of the AP-1 family form homo- and heterodimeric transcription factor complexes with distinct activating and repressing functions [29]. Additionally, our data suggest that BACH2 may play a notable role in regulating the TNF response of FLS in RA. Like AP-1, BACH2 belongs to the basic region leucine zipper (bZIP) family, but has a slightly different DNA sequence binding site (TGCTGAGTCA) and has a bric-a-brac-tramtrack-broad-complex (BTB) domain, which specifically interacts with co-repressors to repress transcription [30]. BACH2 is a highly conserved repressor with a central function in terminal differentiation, maturation, and activity of B and T cells [31]. Intronic SNPs within the *BACH2* gene have been associated with the risk of different immune-mediated diseases, including RA [32, 33]. Furthermore, de novo motif discovery indicated a potential role for developmental transcription factors of the homeobox and forkhead box protein families in transcriptional repression of genes in TNF-stimulated FLS. Since some of these transcription factors are exclusively expressed in FLS at distal joint locations (HOXA13, HOXD13) [8], this suggests that TNF responses of FLS may be different at specific joint locations. However, de novo DNA motif discovery algorithms have some pitfalls. Due to the non-random nature of the genomic sequence, the rate of false positives is high and partial overlap of enriched motifs with known transcription factor binding motifs may be coincidental. Thus, these results should be considered with caution.

With our approach, we were able to assign RA association signals to immune and/or stromal cells. Genes implicated in T cells, but not in FLS, showed a pattern of involvement in “canonical” T cell immunity, including *CTLA4, CD28, IL2RA,* and *GATA3*. Similarly, genes enriched in B cell-specific enhancers were involved in B cell biology, including *IRF8, BLK*, and *TAB1*. The stromal activation observed in RA joints was clearly reflected in the predicted function of the identified FLS-specific regulatory variants, many of which were previously associated with RA pathogenesis and are connected by functional networks. For example, several genes linked to RA credible SNPs in FLS were implicated in cell proliferation and tumor development (e.g., SPRED2 [34], GRHL2 [35], CDK6 [36], RUNX1 [37], and ZFP36L [38]). The transcription factor ZFP36L negatively regulates the expression of CDK6 [39] by binding to the 3′UTR region of the *CDK6* gene, which contains the credible set SNPs at this locus. CDK6 in turn interferes with DNA binding of Runx1 [40]. Furthermore, CD40 activation in FLS increased the expression of several cytokines relevant in RA, including VEGF and RANKL [41, 42]. RBPJ, a regulatory transcription factor of the Notch signalling pathway, has been shown to repress the activation of CD40 [43]. Similarly, TRAF1 can negatively regulate CD40 activity [44].

Most notably, the association signals on chromosome 6 (*TNFAIP3/IFNGR1*) and chromosome 2 (*IFNAR/IFNGR2*) could critically impact the contribution of FLS to the development of RA. Chromatin interactions in these regions connected RA risk variants with several genes encoding the subunits of type I (*IFNAR1, IFNAR2*), type II (*IFNGR1, IFNGR2*), and type III (*IL-10RB*) interferon receptors in FLS. Furthermore, they tightly linked the IFN response to TNF stimulation by interconnection with the *TNFAIP3* gene, encoding the TNF signalling repressor A20, and by their reaction to TNF stimulation. All three types of interferons signal via the JAK-STAT signalling pathway, which, along with TNF, is one of the central therapeutic targets in RA. IFN pathways are strongly associated with the pathogenesis of RA and IFN-responsive genes are induced in FLS upon stimulation with TNF [45]. A type I interferon gene signature is detectable in up to two thirds of patients with RA [46], and it associates with an increased risk of developing RA as well as with therapeutic response to biological DMARDs like TNF inhibitors [47, 48]. In FLS, TNF induces an extensive interferon gene response via secondary autocrine production of IFNβ and the activation of the IRF1-IFNβ-IFNAR-JAK-STAT1 axis [49, 50]. Down syndrome (trisomy 21) leads to increased dosage of the IFN receptors encoded on chromosome 21, which results in a type I interferon gene signature with constant activation of interferon pathways in fibroblasts [51]. Notably, people with Down syndrome are at increased risk of developing erosive, inflammatory seronegative arthritis of their hands and wrists [52]. Together, this strongly suggests a causal role for stromal activation of IFN pathways in the development of RA.

We showed that RA risk allele rs874040 is associated with reduced expression of *RBPJ* in FLS in a location-specific manner. RBPJ, also called CBF1 or CSL, is a key transcriptional regulator of the Notch signalling pathway [53]. In the absence of Notch signalling, RBPJ represses Notch target genes (e.g., HES1). Upon activation of Notch signalling, RBPJ binds to the intracellular domain of the Notch receptor and enhances Notch-dependent gene expression. Loss of RBPJ leads to activation of dermal fibroblasts and promotes their transformation into cancer-associated fibroblasts (CAFs), which play a crucial role in tumor development and growth [54, 55]. Activation of Notch signalling was shown in RA FLS and induced FLS proliferation [56]. Furthermore, Notch signalling is critical for shaping the synovial environment by guiding the development of THY1+ sublining FLS, a subset of FLS that is expanded in RA synovial tissues [57]. Constitutive lower levels of RBPJ in FLS from individuals carrying the RBPJ risk variant could favor synovial enrichment of THY1+ sublining FLS, which are considered critical for the development of RA. Joint-specific differences in the chromatin landscape in this locus exemplify how genetic risk could result in the specific patterns of joint involvement that typically occur in chronic inflammatory joint diseases. Additionally, joint-specific expression of HOX transcription factors [8], for which we suggest a role in gene repression after TNF stimulation in FLS, could contribute to joint-specific differences in the susceptibility to RA.

Further pathways that we found enriched in genes targeted by RA credible SNPs were connected to lipid metabolism and the primary cilium. FADS1 and 2 have been implicated in the production of anti-inflammatory unsaturated fatty acids in LPS-treated macrophages, contributing to the resolution phase of LPS-driven inflammatory response in macrophages [58]. Changes in the lipid metabolism have been suggested in RA FLS [59], but specific functional data does not exist so far. The primary cilium serves as a hub for several cell signalling pathways, e.g., Notch [60] and wnt signalling [61]. In FLS, it was shown that TNFR1 and TNFR2 localize to the cilium pit [62]. The cilium connected proteins C5orf30 and GSN that we found interacting with RA risk variants in FLS were previously shown to be negative regulators of arthritis in mice [63, 64]. Another ciliary protein, SPAG16, was found to be a genetic risk factor for joint damage progression in RA patients, increasing the production of matrix-metalloproteinases in FLS [65]. Future studies are required to demonstrate how changes in lipid metabolism and primary cilium affect the function of FLS and influence RA pathogenesis.

With our approach, we could connect several RA association signals with potential pathogenic genes and pathways in RA FLS. However, our data does not allow any conclusion on differences in the chromatin landscape that might exist between healthy FLS and RA FLS as previously shown [66]. Therefore, we cannot exclude the possibility that there are differences in chromatin interactions and open chromatin between healthy and RA FLS that we cannot detect in our study and that would affect our results. However, the epigenetic landscape in RA FLS might already be changed before the onset of the disease, as shown for changes in DNA methylation [67] and this might be one reason why these genetic risk factors trigger the disease in some people but not in others. To address this, longitudinal studies analyzing the epigenetic landscape at different stages of disease development are needed. Another limitation of our study is the low samples size used in most experiments, which again is due to the more difficult accessibility of synovial tissues compared to, e.g., blood. The statistical analysis must therefore be interpreted with caution.

## Conclusions

Overall, our research significantly advances the knowledge about putative causal SNPs, enhancers, genes, and cell types affected by genetic risk loci in RA. Our analysis can direct future studies to investigate pathways that are genetically affected in a cell-type-specific way. This will ultimately enable the connection of an individual’s genetic risk with the causal pathways and cell types that drive disease, paving the way to stratified treatment decisions and precision medicine.

## Methods

### Patients and cell culture

Synovial tissues were obtained from OA and RA patients undergoing joint replacement surgery at the Schulthess Clinic Zurich, Switzerland. Patient’s characteristics are described in Additional file 1: Table S1. RA patients fulfilled the 2010 ACR/EULAR (American College of Rheumatology/European League Against Rheumatism) criteria for the classification of RA [68]. Samples from patients with joint pain without inflammation or cartilage destruction (healthy, 3 male/3 female, mean age 39, range 23–49) were obtained from the Queen Elizabeth Hospital in Birmingham, UK. Synovial tissues were digested with dispase (37 °C, 1 h) and FLS were cultured in Dulbecco’s modified Eagle’s medium (DMEM; Life Technologies) supplemented with 10% fetal calf serum (FCS), 50 U ml^−1^ penicillin/streptomycin, 2 mM l-glutamine, 10 mM HEPES, and 0.2% amphotericin B (all from Life Technologies). Purity of FLS cultures was confirmed by flow cytometry showing the presence of the fibroblast surface marker CD90 (Thy-1) and the absence of leukocytes (CD45), macrophages (CD14; CD68), T lymphocytes (CD3), B lymphocytes (CD19), and endothelial cells (CD31). Cell cultures were negative for mycoplasma contamination as assessed by MycoAlert mycoplasma detection kit (Lonza). FLS were used between passages 4–8. Information on the assays performed on each sample is given in Additional file 2: Table S2.

### RNA sequencing

RNA sequencing data from unstimulated samples (Additional file 2: Table S2) was retrieved from the European Nucleotide Archive (ENA) with the primary accession code PRJEB14422. A detailed description of sample preparation and sequencing procedures is given in Frank-Bertoncelj et al. [8]. For RNA sequencing of TNF-stimulated FLS, cultured FLS were treated with 10 ng/ml human recombinant TNF (Roche) for 24 h or were left untreated. Total RNA was isolated using the RNeasy Mini kit (Qiagen) including on-column DNAase I digestion. Part of the libraries (*n* = 12) were prepared using the NEB Next Ultra Directional RNA-seq protocol with ribosomal depletion and were sequenced using Illumina HiSeq4000 with 75 bp paired end reads. The additional libraries (*n* = 20) were generated using the Illumina TruSeq Stranded total RNA protocol with the TruSeq Stranded total RNA Sample Preparation Kit and were sequenced using Illumina Novaseq 6000. All Fastq-files were mapped to hg19 and sequence reads assigned to genomic features using STAR [69] and featureCounts [70], respectively. We used svaseq R [71] package (version 3.36.0) to find and remove hidden batch effects. Differential gene expression analysis was performed with DESeq2 [72] R package (version 1.28.1) according to standard protocol. The normalization was performed as part of the standardized DESeq2 workflow (applying the concept of variance stabilizing transformations (VST) [73, 74].

### ChIP sequencing

ChIP DNAseq was performed on the Illumina HiSeq 2500 (50 bp, single end) as described in Frank-Bertoncelj et al. [8]. Briefly, ChIP assays were performed using 1 million FLS (Additional file 2: Table S2) per IP and the following antibodies (all Diagenode): H3K4me3 (0.5 μg, C15411003), H3K27me3 (1 μg, C15410195), H3K27ac (1 μg, C15410196), H3K4me1 (1 μg, C15410194), H3K36me3 (1 μg, C15410192), and H3K9me3 (1 μg, C15410193). The reads were mapped to the GRCh38 human genome reference using Bowtie2 [75] with default settings. The mapped alignment files were further QC’ed with Picard Tools (Broad Institute, available at: http://broadinstitute.github.io/picard/) to check for duplication rates, unique mapping reads, and library complexity. The duplicated reads and non-unique mapping reads were then removed prior to analysis with Picard Tools.

### ChromHMM chromatin state inference

The de-duplicated, uniquely mapping reads of the ChIP sequencing were binarized with the BinarizeBam script provided by the chromHMM software [13]. This script splits the genome into 200 bp bins and compares the coverage of the alignment file at each bin with the input sequence file to determine if any histone modification is present in the bin (1 = yes, 0 = no). The pre-trained 18 state chromHMM model based on the six histone marks was applied to the binarized bed files, using the MakeSegmentation script provided and the model parameters downloaded from the Roadmap Epigenomics web portal. The methods employed by Ernst et al. [13] were replicated where possible from the data processing stages to the chromatin state inference.

### ATAC sequencing

Cultured RA FLS were stimulated with 10 ng/ml TNF for 24 h or were left untreated (Additional file 2: Table S2). From each patient cell line, 50'000 cells were prepared according to the protocol by Buenrostro et al [76]. ATAC-seq libraries were sequenced on Illumina HiSeq 4000 with 75 bp paired end reads. The reads were QC’d with FastQC for read quality, and the Nextera-transposase adaptors were trimmed with cutadapt [77]. The reads were aligned with Bowtie 2 to the GRCh38 human reference. PCR duplicates were identified and removed by Picard Tools prior to peak calling using MACS2 [78]. Both broad and narrow peaks were called as ATAC-seq can have properties of both.

### HiC and capture HiC

Cultured human FLS from RA patients were treated with 10 ng/ml TNF for 24 h or were left untreated (Additional file 2: Table S2). Cells (1–3 × 10^7^ per condition), were scratched in 10 ml DMEM, spun down, suspended in 35 ml DMEM, and fixed (2% formaldehyde in DMEM, 10 min, RT, with mixing on a rocker). The reaction was quenched with cold 0.125 M glycine. Cells were incubated at RT for 5 min, followed by 15 min incubation on ice and centrifugation (1500 rpm, 10 min, 4 °C). Pellets were suspended and washed in cold PBS (1500 rpm, 10 min, 4 °C). Washed pellets were snap frozen and stored at − 80 °C. HiC libraries from RA FLS samples were generated as previously described [14]. They were sequenced on Illumina HiSeq 4000 with 75 bp paired end reads. The reads were processed using the HiC Pro pipeline [79], and the correlation between samples were calculated with HiCrep [80].

TADs were called with TADCompare [15]. The regions targeted by the capture HiC (CHiC) were generated based on the LD regions of the lead disease-associated SNPs for RA, juvenile idiopathic arthritis (JIA), psoriatic arthritis (PsA), and psoriasis (Ps). This resulted in a total of 242 distinct risk variants. Then, 120 bp capture baits were designed for all HindIII digestion fragments overlapping these regions as previously described in Martin et al. [4]. Significant CHiC interactions were identified through the CHiCAGO pipeline [81], where the suggested threshold of CHiCAGO score > 5 was used. Differential interactions were identified with DESeq2, where the read counts of each interaction were treated similar to the gene count of RNA-seq.

### Transcription factor binding site prediction

We extracted differentially interacting regions from our CHiC data, where the strength in chromatin interaction (log-fold change of read counts between basal and stimulated) correlated with nearby genes. We overlapped these interaction regions (bait and prey fragments) with our ATAC-seq peaks. These ATAC-seq peaks were standardized and re-centered to 200 bp each. We then used the findMotifsGenome.pl software from the HOMER suite [82] to identify significantly enriched motifs in these ATAC-peaks compared to random background sequences chosen by HOMER.

### Partitioned heritability

We defined active chromatin regions of the genome for each FLS sample and publicly available Roadmap samples, based on the union of H3K27ac, H3K4me1, and H3K4me3 histone peaks. We used the partitioned heritability software from the LDSC [17] package to quantify the non-HLA RA heritability attributed to these active regions in each sample, based on the summary statistics from the Okada et al. trans-ethnic meta-analysis [2].

### Derivation of RA credible set SNPs

For each locus, we dissected distinct RA association signals using approximate conditioning implemented in GCTA [18], based on (i) European ancestry summary statistics from the Okada et al. trans-ethnic meta-analysis [2]; and (ii) a reference panel of European ancestry haplotypes from the 1000 Genomes Project to approximate linkage disequilibrium between SNPs. We identified index SNPs for each distinct signal, at a locus-wide significance threshold of *p* < 10^−5^, using the --cojo-slct option. For each locus with multiple distinct signals, we derived the conditional association summary statistics for each distinct signal by conditioning out the effects of all other index SNPs at the locus using the --cojo-cond option.

For each distinct signal, we first calculated the posterior probability, *π*_*j*_, that the *j*th variant is driving the association, given by

$$ {\pi}_j=\frac{\varLambda_j}{\sum_k{\varLambda}_k}, $$

where the summation is over all variants at the locus. In this expression, *Λ*_*j*_ is the approximate Bayes’ factor [83] for the *j*th variant, given by

$$ {\varLambda}_j=\sqrt{\frac{V_j}{V_j+\omega }}\exp \left[\frac{\omega {\beta}_j^2}{2{V}_j\left({V}_j+\omega \right)}\right], $$

where *β*_*j*_ and *V*_*j*_ denote the estimated allelic effect and corresponding variance from the European ancestry component of Okada et al. [2]. In loci with multiple distinct signals of association, summary statistics were obtained from the approximate conditional analysis. In loci with a single association signal, summary statistics were obtained from unconditional analysis. The parameter *ω* denotes the prior variance in allelic effects, taken here to be 0.04. The 99% credible set for each signal was then constructed by (i) ranking all variants according to their Bayes’ factor, *Λ*_*j*_; and (ii) including ranked variants until their cumulative posterior probability of driving the association attained or exceeded 0.99.

### Pathway analysis and protein-protein interaction network

The genes assigned to FLS (Additional file 8: Table S5, column R) and listed in Additional file 8: Table S5, columns U-Z were analyzed by STRINGv11 (interactions settings to medium confidence levels) [84], ToppFun on ToppGene Suite [85], and DAVID v6.8 [21, 22] with default settings.

### Luciferase reporter assay

Single-stranded oligonucleotides corresponding to 31 nucleotide fragments of the human genome with the variant in the middle including a BamHI and SalI restriction site were purchased (Microsynth). Double-stranded oligonucleotides were generated by mixing equal amounts of complementary oligonucleotides and incubated in a thermocycler for 5 min at 95 °C and then slowly cooled to room temperature (− 1 °C/min). Double-stranded oligonucleotides were cloned downstream from the luciferase gene in the pGL3-promoter vector (Promega). 8 × 10^4^ HT1080 cells were transfected with 1 μg of the pGL3-promoter vector together with 0.1 ng of the pRL-SV40 vector (Promega) using 1.5 μl of Lipofectamine 2000 (Invitrogen). After 18 h, firefly and renilla luciferase activity was measured using the Dual-Glo Luciferase Assay System (Promega). Firefly luciferase activity was corrected for renilla luciferase activity and the data were normalized to cells transfected with the empty pGL3-promoter vector.

### Guide RNA design and cloning

Guide (g)RNAs, targeting the putative upstream *RBPJ* enhancer (locus 23, Additional file 8: Table S5), were designed using the CRISPOR tool [86] in the DNA region chr4:26106475-26106675 (hg38) comprising 100 bp upstream and 100 bp downstream of the RA risk SNP rs874040 (gRNA_9: 5′ GCCTTATCATGGCATATCACC 3′; PAM TGG; gRNA_12: 5′ GCTAGAGCACGCAGCTTTTGC 3′; PAM AGG). Complementary gRNA oligo pairs with 5′ CACC (fwd) and 5′CAAA (rev) overhangs (Microsynth, 100 mM) were phosphorylated and annealed in a thermocycler (37 °C, 30 min; 95 °C 5 min, ramp down to 25 °C at 5 °C/min using T4PNK (NEB) and 10× T4 ligation buffer (NEB). LentiGuide-puro plasmid, a gift from Feng Zhang (Addgene μplasmid # 52963; http://n2t.net/addgene:52963; RRID:Addgene_52963 )[87], was digested with FastDigest BBsI, Fast AP, 10× Fast Digest Buffer at 37 °C, 30 min (Fermentas) followed by the ligation of the annealed gRNA duplex o/n using Quick Ligase (NEB) and 2× Quick Ligation buffer (NEB). One Shot™ Stbl3™ Chemically Competent *E. coli* (Thermo Fisher, C7373-03) were transformed with gRNA-containing lentiGuide-Puro plasmids by heat-shocking (45 s, 42 °C) according to the manufacturer’s instructions. Plasmid DNA from selected colonies was isolated using QIAprep Spin Miniprep Kit (Qiagen) and Sanger sequenced to confirm the insertion and sequences of cloned gRNAs. To prepare gRNA-containing lentiviral particles, HEK293T cells were transfected with psPAX2, pMD2.G gRNA-containing plasmids (total 10 μg plasmid DNA, mass ratio 2:1:4, respectively). psPAX2 (Addgene plasmid # 12260; http://n2t.net/addgene:12260; RRID: Addgene_12260) and pMD2.G (Addgene plasmid # 12259; http://n2t.net/addgene:12259; RRID: Addgene_12259) were a gift from Didier Trono. Viral particles were precipitated from the supernatants of transfected HEK293T (24 h and 48 h) using PEG-itTM Virus Precipitation Solution (5×) according to the manufacturer’s protocol (System Biosciences), resuspended in PBS, and stored at − 70 °C.

### Activation of enhancer regions with dCas9-VPR

FLS were transduced with Edit-R Lentiviral dCas9-VPR lentiviral particles (hEF1α promoter, Dharmacon). Edit-R Lentiviral dCas9-VPR is a CRISPR activation system, in which a nuclease-deactivated *S. pyogenes* Cas9 (dCas9) is fused to VP64, p65, and Rta transcriptional activators. Stable populations of dCas9-VPR FLS were blasticidin selected (7.5 μg/ml, Horizon) and subsequently transduced with gRNA-containing lentiviral particles. Stable gRNA dCas9-VPR FLS were puromycin selected (5 μg/ml Sigma) and lysed and RNA was isolated, followed by reverse transcription and SYBR Green real-time PCR as described above. Gene expression was normalized to the average expression of *B2M* (Primer sequence Fwd 5′ AAGCAGCATCATGGAGGTTTG 3′, Rev 5′ AAGCAAGCAAGCAGAATTTGGA 3′) and *RPLP0* housekeeper genes. Transduction of dCas9-VPR without guide RNA had no effect on *RBPJ* expression.

### Pyrosequencing

DNA from FLS was isolated using the QIAamp DNA Blood kit (Qiagen). DNA regions containing rs874040 (*RBPJ*) were amplified by PCR (Primers: Fwd 5' AGTGTGGATTGTAGCAGATATGTC 3'; Rev biotin- 5' ACCAAGGCAGCCACAGAATC 3'; 5' GCTCGGATGGGGTATTTC TAG 3'). SNPs were genotyped by pyrosequencing using PyroMark Q48 Advanced Reagents and the PyroMark Q48 Autoprep (both Qiagen) according to the manufacturer’s instructions.

### Quantitative real-time PCR

Total RNA was isolated using the RNeasy Mini Kit (Qiagen) and the Quick RNA MicroPrep Kit (Zymo Research) including on-column DNaseI digest and was reverse transcribed. SYBRgreen real-time PCR was performed (primers: *RBPJ* Fwd 5' GGCTGCAGTCTCCACGTACGTC 3', Rev 5' CTCACCAAATTTCCCAGGCGATGG 3'; *HES1* Fwd 5' ATGGAGAAAAGACGAAGAGCAAG 3'; Rev 5' TGCCGCGAGCTATCTTTCTT 3'), including controls (samples containing the untranscribed RNA, dissociation curves). Data were analyzed with the comparative CT methods and presented as ΔCT or 2^−ΔΔCT^ as described elsewhere [88] using *RPLP0* as a housekeeping gene for sample normalization (Fwd 5' GCGTCCTCGTGGAAGTGACATCG 3', Rev 5' TCAGGGATTGCCACGCAGGG 3').

### Cap Analysis Gene Expression (CAGE)

Cultured human FLS from RA patients were treated with 10 ng/ml TNF, 24 h, or were left untreated (Additional file 2: Table S2). RNA was isolated using the Quick RNA MicroPrep Kit (Zymo Research). CAGE libraries were prepared and sequenced as previously described in detail [89]. Mapping and identification of CAGE transcription start sites (CTSSs) were performed by DNAFORM (Yokohama, Kanagawa, Japan). In brief, the sequenced CAGE tags were mapped to hg19 using BWA software and HISAT2 after discarding ribosomal RNAs. Identification of CTSSs was performed with the Bioconductor package CAGEr (version 1.16.0) [90]. TSS and enhancer candidate identification and quantification were performed with the Bioconductor package CAGEfightR (version 1.6.0) [91] with default settings.

## Supplementary Information

**Additional file 1:.** Table S1. Clinical data on patient’s samples.

**Additional file 2:.** Table S2. Information on methods applied to patient’s samples.

**Additional file 3:.** Dataset S1. Quality control measures.

**Additional file 4:.** Dataset S2. Known motif enrichment analysis using HOMER at enhancer sites with open chromatin.

**Additional file 5:.** Figures S1 – S7.

**Additional file 6:.** Table S3. Definition of RA loci and number of distinct association signals attaining locus-wide significance (p < 10^-6^) in European ancestry GWAS meta-analysis.

**Additional file 7:.** Table S4. Distinct signals of association attaining locus-wide significance (p < 10^-6^) in European ancestry GWAS meta-analysis and corresponding 99% credible SNP sets.

**Additional file 8:.** Table S5. Detailed information on the 73 loci

**Additional file 9:.** Review history

## Data Availability

RNA sequencing data are available in the European Nucleotide Archive (ENA) accession PRJEB14422 https://www.ebi.ac.uk/ena/browser/view/PRJEB14422 [8]. The Capture HiC data from GM12878 and Jurkat cell lines are available in the NCBI Gene Expression Omnibus (GEO) repository accession GSE69600 https://www.ncbi.nlm.nih.gov/geo/query/acc.cgi?acc=GSE69600 [4]. The RNA-seq datasets from stimulated FLS, the ChIP-seq datasets, the ATAC-seq datasets, the Capture HiC datasets, and the CAGE dataset are available in the GEO repository accession GSE163548 https://www.ncbi.nlm.nih.gov/geo/query/acc.cgi?acc=GSE163548 [92].
