# Supplementary material for: Functional genomics atlas of synovial fibroblasts defining rheumatoid arthritis heritability
Source: Genome Biol. 2021 Aug 25;22:247. doi: 10.1186/s13059-021-02460-6 (PMC8385949; doi:10.1186/s13059-021-02460-6)
Supplement: Supplementary file 3 — Additional file 3:. Dataset S1. Quality control measures. [file 13059_2021_2460_MOESM3_ESM.pdf]

Dataset S1.

a) Quality check results of the ChIP-seq dataset.

	H3K4me1				H3K4me3				H3K9me3			
**Sample ID**	**Total Sequences (M)**	**Unmapped reads (M)**	**PERCENT DUPLICATION (%)**	**VALID READS (M)**	**Total Sequences (M)**	**Unmapped reads (M)**	**PERCENT DUPLICATION (%)**	**VALID READS (M)**	**Total Sequences (M)**	**Unmapped reads (M)**	**PERCENT DUPLICATION (%)**	**VALID READS (M)**
**88**	32.8	0.5	43.8	18.2	38.7	1.04	56.7	16.3	67.9	1.45	22.2	51.7
**316**	41.4	0.5	38.7	25.1	31.9	0.32	30.2	22	87.6	1.78	25.7	63.8
**335**	48.2	0.7	49.8	23.8	29.7	0.43	33.2	19.6	74.1	2.36	40.1	43
**341**	42.2	0.6	40.6	24.7	29.4	0.56	43	16.4	66.5	1.49	19.1	52.6
**343**	43.5	0.6	51.6	20.8	29.8	0.47	28.9	20.9	49.2	1.1	19.2	38.9
**368**	51.6	0.5	54.6	23.2	37.7	0.61	45.2	20.3	61.4	1.52	43.3	34
**384**	37.4	0.8	62	13.9	30.1	0.42	32	20.2	59.3	1.24	22	45.3

	H3K27ac				H3K27me3				H3K36me3			
**Samples**	**Total Sequences (M)**	**Unmapped reads (M)**	**PERCENT DUPLICATION (%)**	**VALID READS (M)**	**Total Sequences (M)**	**Unmapped reads (M)**	**PERCENT DUPLICATION (%)**	**VALID READS (M)**	**Total Sequences (M)**	**Unmapped reads (M)**	**PERCENT DUPLICATION (%)**	**VALID READS (M)**
**88**	40.4	0.5	37.9	24.8	48.4	2.81	78.4	9.8	62.5	0.55	15.2	52.5
**316**	39.7	0.39	28.4	28.1	53.1	0.69	52.2	25.1	64.4	0.53	18.9	51.8
**335**	44.6	0.83	52.3	20.9	51.3	0.76	64.9	17.7	84.2	1	21.8	65.1
**341**	35.8	0.62	54.9	15.9	52.6	0.71	52.6	24.6	70.2	0.97	22.6	53.6
**343**	36.9	0.51	44.5	20.2	52	0.87	58.8	21.1	50	0.61	18	40.5
**368**	45.5	0.91	52.6	21.1	51.5	0.74	56.7	22	63.4	0.87	28.7	44.6
**384**	47.1	1.5	80.1	9.1	49.3	0.86	59.5	19.6	68.3	0.63	16.3	56.6

b) Hi-C read information and quality checks.
Samples are unstimulated controls, or have been TNF stimulated (_TNF rows).

Sample ID	Joint	Total Reads (M)		Unique Alignments (M)		Paired Reads (M)	Valid Pairs (M)	Percentage Mapped (%)
		R1	R2	R1	R2			
**SF_412**	Wrist	103	103	80	79	63	51	61.85
**SF_412_TNF**	Wrist	110	110	88	87	71	61	64.88
**SF_415A**	Wrist	112	112	84	83	64	48	57.2
**SF_415A_TNF**	Wrist	591	591	467	463	371	347	62.91
**SF_415B**	MCP (Hand)	56	56	41	41	30	20	54.31
**SF_415B_TNF**	MCP (Hand)	96	96	76	75	60	51	62.16
**SF_420**	Shoulder	120	120	94	92	72	63	60.64
**SF_420_TNF**	Shoulder	291	291	230	222	174	154	59.95
**SF_424**	MCP (Hand)	79	79	63	62	51	46	64.63
**SF_424_TNF**	MCP (Hand)	83	83	67	66	54	48	64.57
**SF_427**	Knee	419	419	321	316	249	170	59.39
**SF_427_TNF**	Knee	277	277	213	209	166	115	60.1
**SF_429**	Shoulder	291	291	236	233	193	164	66.26
**SF_429_TNF**	Shoulder	301	301	245	241	201	163	66.95

c) Capture Hi-C read information and quality checks.
Samples are unstimulated controls or have been TNF stimulated (_TNF rows).

Sample ID (capture Hi-C)	Joint	Total Reads (M)		Unique Alignments (M)		Paired Read (M)	Valid Pairs (M)	Percentage Mapped (%)
		R1	R2	R1	R2			
**SF_412**	Wrist	203	203	158	155	125	104	61.8
**SF_412_TNF**	Wrist	332	332	266	262	218	189	65.73
**SF_415A**	Wrist	247	247	187	186	143	124	57.93
**SF_415A_TNF**	Wrist	155	155	123	122	100	94	64.55
**SF_415B**	MCP (Hand)	336	336	239	237	173	122	51.7
**SF_415B_TNF**	MCP (Hand)	310	310	250	247	200	172	64.78
**SF_420**	Shoulder	217	217	177	175	143	127	65.98
**SF_420_TNF**	Shoulder	156	156	128	126	103	92	66.05
**SF_424**	MCP (Hand)	173	173	144	141	117	105	67.52
**SF_424_TNF**	MCP (Hand)	154	154	126	125	104	95	67.51
**SF_427**	Knee	218	218	161	159	124	92	56.84
**SF_427_TNF**	Knee	216	216	167	164	131	99	60.6
**SF_429**	Shoulder	216	216	175	173	145	124	67.11
**SF_429_TNF**	Shoulder	252	252	206	202	171	141	68.04

d) ATAC-seq read information and quality checks.
Samples are unstimulated controls, or have been TNF stimulated (_TNF rows).

Sample ID (ATAC)	READ PAIRS EXAMINED (M)	READ PAIR DUPLICATES (M)	PERCENT DUPLICATION (%)	ESTIMATED LIBRARY SIZE (M)
**SF_346**	94.3	68.5	72.7	26.5
**SF_346_TNF**	85.3	37.7	44.1	66.4
**SF_415B**	75.1	47.9	63.8	29.6
**SF_415B_TNF**	73.7	27	36.7	75.5
**SF_420**	78.9	57.1	72.3	22.5
**SF_420_TNF**	67.3	54	80.3	13.3
**SF_424**	72.8	59.2	81.3	13.7
**SF_424_TNF**	90.1	59.3	65.8	33.1
**SF_427**	81.6	55.1	67.5	28.1
**SF_427_TNF**	63.3	42.7	67.5	21.8
**SF_429**	95.3	59.9	62.8	38.8
**SF_429_TNF**	65.8	32.7	49.7	41.9

e) RNA-seq data quality and alignment summary.
Samples are unstimulated controls or have been TNF stimulated (_TNF rows).

Sample ID	Total bases (M)	Aligned bases (M)	Coding bases (%)	UTR bases (%)	Intronic bases (%)	Intergenic bases (%)	Usable bases (%)
**SF_292**	10092.8	9943.9	16.7	18.8	27.3	37.2	35
**SF_292_TNF**	7794.8	7673.9	10.6	16.2	27.2	46.1	26.3
**SF_336**	8206.8	8072	10.4	20.3	25.5	43.8	30.2
**SF_336_TNF**	7983.2	7846.4	12.6	18.8	26.6	42	30.9
**SF_346**	7263.5	7130.7	9.9	16.2	27.3	46.6	25.6
**SF_346_TNF**	6830.9	6705.5	11.3	18.4	26.6	43.6	29.2
**SF_407**	6360.5	6251	20.4	20.5	24.5	34.6	40.2
**SF_407_TNF**	8051.5	7954.8	6.7	17.1	28.1	48.1	23.5
**SF_415B**	4927.7	4858.9	6	16.2	27.6	50.2	22
**SF_415B_TNF**	8369.4	8271.4	6.2	17.1	28.8	47.9	22.9
**SF_435**	8165.7	8052.1	5.2	15.9	27	52	20.7
**SF_435_TNF**	10682.4	10529.9	6	17	29.2	47.7	22.7

f) CAGE-seq data quality and alignment summary.
Samples are unstimulated controls or have been TNF stimulated (_TNF rows).

Sample ID	Total Reads (M)	rRNA-Filtered (M)	BWA-Mapped (M)	HISAT2-Mapped (M)	Total Mapped (M)	Percentage Mapped (%)
**SF_276**	25.8	8.7	14.3	1.7	16.0	62.1
SF_276_TNF	25.4	7.6	15.2	1.4	16.6	65.5
**SF_415**	24.9	4.7	17.7	1.6	19.3	77.6
**SF_415_TNF**	20.7	4.3	13.9	1.4	15.3	74.2
**SF_420**	25.0	6.2	16.0	1.5	17.4	69.8
SF_420_TNF	25.3	5.6	17.0	1.5	18.5	73.0
SF_424	24.9	7.6	14.6	1.7	16.4	65.8
SF_424_TNF	27.5	14.2	10.6	1.6	12.2	44.5
**SF_427**	24.3	7.5	14.1	1.6	15.7	64.6
**SF_427_TNF**	23.6	5.9	15.2	1.4	16.6	70.2
**SF_429**	25.6	7.7	15.0	1.8	16.8	65.8
**SF_429_TNF**	25.0	5.2	17.3	1.6	18.9	75.5
**SF_460**	20.8	4.8	13.8	1.4	15.2	73.1
**SF_460_TNF**	24.0	4.7	16.5	1.6	18.1	75.6

BWA = Burrows-Wheeler Aligner
