# Supplementary material for: Functional genomics atlas of synovial fibroblasts defining rheumatoid arthritis heritability
Source: Genome Biol. 2021 Aug 25;22:247. doi: 10.1186/s13059-021-02460-6 (PMC8385949; doi:10.1186/s13059-021-02460-6)
Supplement: Supplementary file 4 — Additional file 4:. Dataset S2. Known motif enrichment analysis using HOMER at enhancer sites with open chromatin. [file 13059_2021_2460_MOESM4_ESM.pdf]

Dataset S2. Known motif enrichment analysis using HOMER at enhancer sites with open chromatin (as defined by ATAC-seq).

STIMULATED ATAC-seq peaks cut-off p-value = 1.00E-04

CHiC up/RNA up

Rank	Motif	Name	P-value	log P-pvalue	q-value (Benjamini)	# Target Sequences with Motif	% of Targets Sequences with Motif	# Back-ground Sequences with Motif	% of Back-ground Sequences with Motif
1	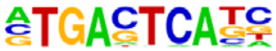	AP-1(bZIP)/ThioMac-PU.1-ChIP-Seq(GSE21512)/Homer	1.00E-12	-2.97E+01	0	45	20.83%	2901.5	5.90%
2	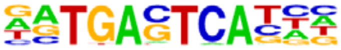	Atf3(bZIP)/GBM-ATF3-ChIP-Seq(GSE33912)/Homer	1.00E-10	-2.35E+01	0	38	17.59%	2576.3	5.24%
3	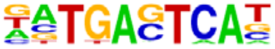	BATF(bZIP)/Th17-BATF-ChIP-Seq(GSE39756)/Homer	1.00E-08	-1.91E+01	0	34	15.74%	2494.7	5.07%
4	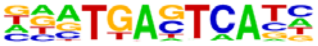	Fra1(bZIP)/BT549-Fra1-ChIP-Seq(GSE46166)/Homer	1.00E-06	-1.58E+01	0	28	12.96%	2061.2	4.19%
5	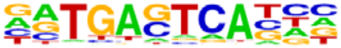	Fosl2(bZIP)/3T3L1-Fosl2-ChIP-Seq(GSE56872)/Homer	1.00E-04	-1.10E+01	0.0011	18	8.33%	1274.9	2.59%

CHiC up/RNA down

Rank	Motif	Name	P-value	log P-pvalue	q-value (Benjamini)	# Target Sequences with Motif	% of Targets Sequences with Motif	# Back-ground Sequences with Motif	% of Back-ground Sequences with Motif
1	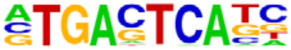	AP-1(bZIP)/ThioMac-PU.1-ChIP-Seq(GSE21512)/Homer	1.00E-24	-5.62E+01	0	76	23.31%	2904.8	5.89%
2	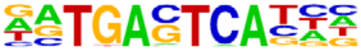	Atf3(bZIP)/GBM-ATF3-ChIP-Seq(GSE33912)/Homer	1.00E-21	-5.02E+01	0	67	20.55%	2511.4	5.09%
3	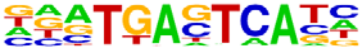	Fra1(bZIP)/BT549-Fra1-ChIP-Seq(GSE46166)/Homer	1.00E-19	-4.52E+01	0	57	17.48%	2008.9	4.07%
4	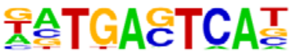	BATF(bZIP)/Th17-BATF-ChIP-Seq(GSE39756)/Homer	1.00E-17	-4.02E+01	0	60	18.40%	2473.5	5.01%
5	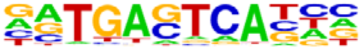	Fosl2(bZIP)/3T3L1-Fosl2-ChIP-Seq(GSE56872)/Homer	1.00E-12	-2.82E+01	0	34	10.43%	1147.5	2.32%
6	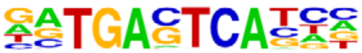	Jun-AP1(bZIP)/K562-cJun-ChIP-Seq(GSE31477)/Homer	1.00E-09	-2.25E+01	0	26	7.98%	846.1	1.71%
7	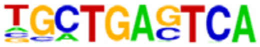	Bach2(bZIP)/OCILy7-Bach2-ChIP-Seq(GSE44420)/Homer	1.00E-04	-1.12E+01	0.0006	16	4.91%	668.4	1.35%
8	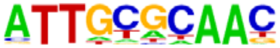	CEBP(bZIP)/ThioMac-CEBPb-ChIP-Seq(GSE21512)/Homer	1.00E-04	-9.37E+00	0.0034	40	12.27%	3191.3	6.47%
