# Supplementary material for: Functional genomics atlas of synovial fibroblasts defining rheumatoid arthritis heritability
Source: Genome Biol. 2021 Aug 25;22:247. doi: 10.1186/s13059-021-02460-6 (PMC8385949; doi:10.1186/s13059-021-02460-6)
Supplement: Supplementary file 5 — Additional file 5:. Figures S1 – S7. [file 13059_2021_2460_MOESM5_ESM.pptx]

## Slide 1
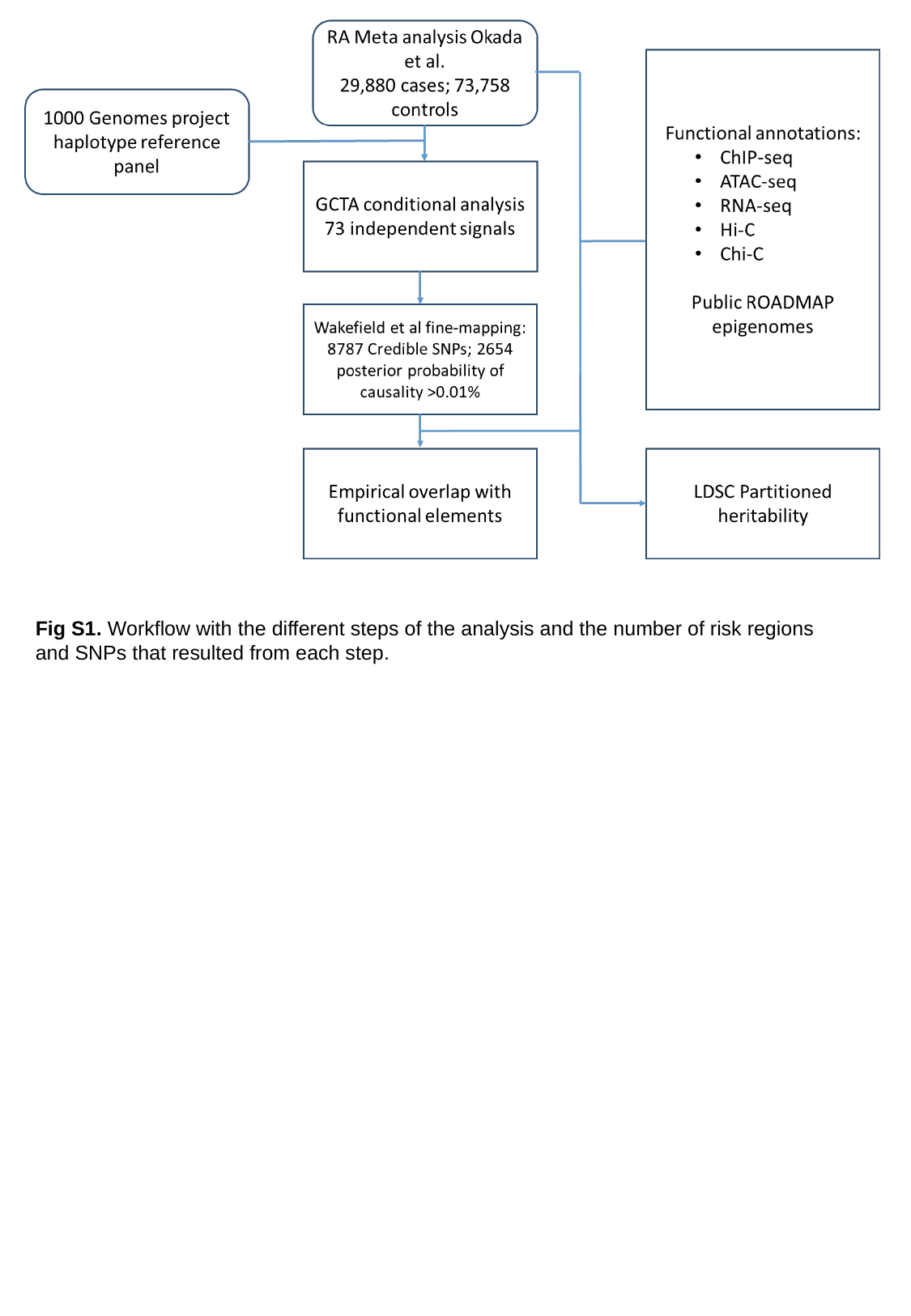

Fig S1. Workflow with the different steps of the analysis and the number of risk regions and SNPs that resulted from each step.

## Slide 2
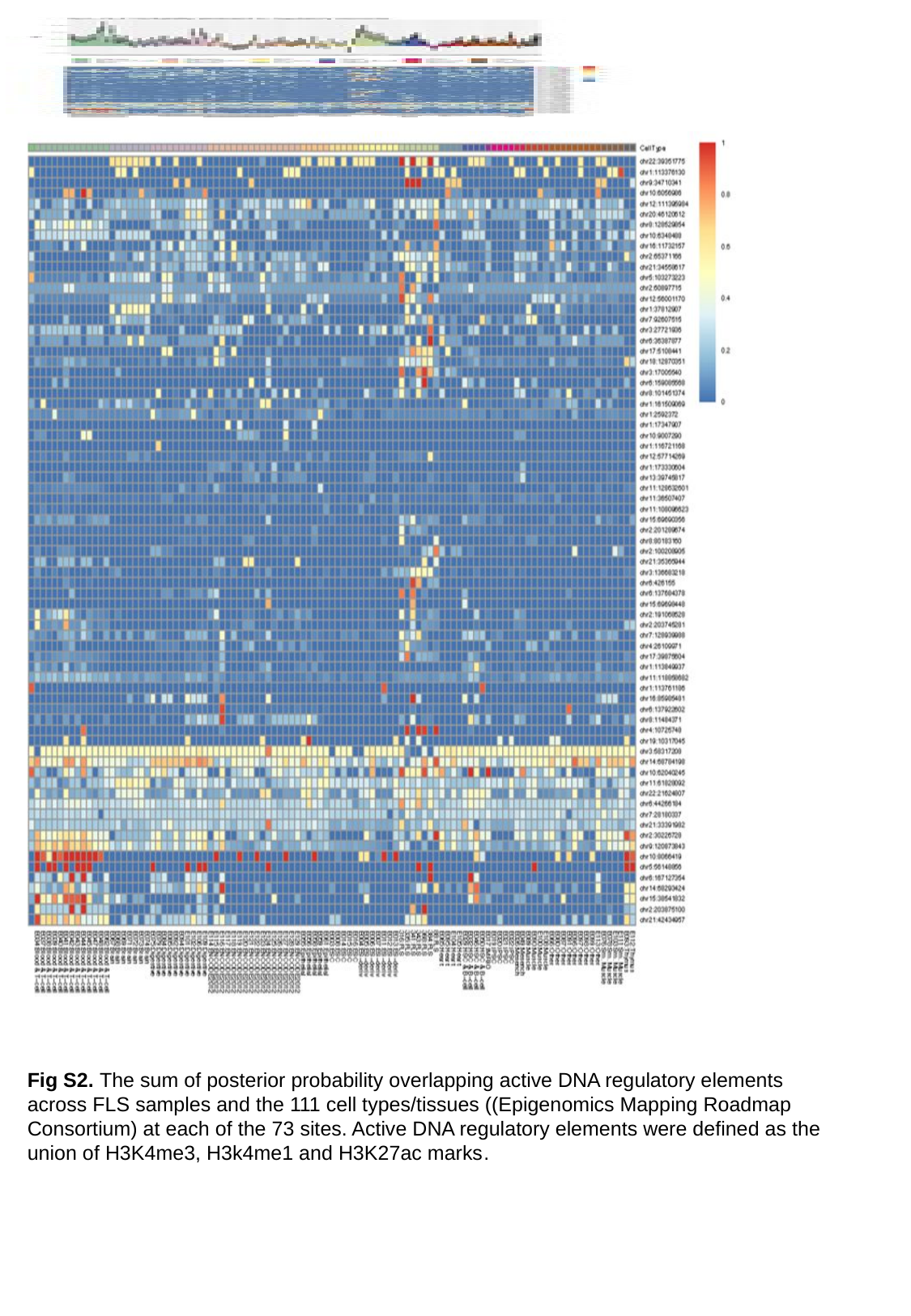

Fig S2. The sum of posterior probability overlapping active DNA regulatory elements across FLS samples and the 111 cell types/tissues ((Epigenomics Mapping Roadmap Consortium) at each of the 73 sites. Active DNA regulatory elements were defined as the union of H3K4me3, H3k4me1 and H3K27ac marks.

## Slide 3
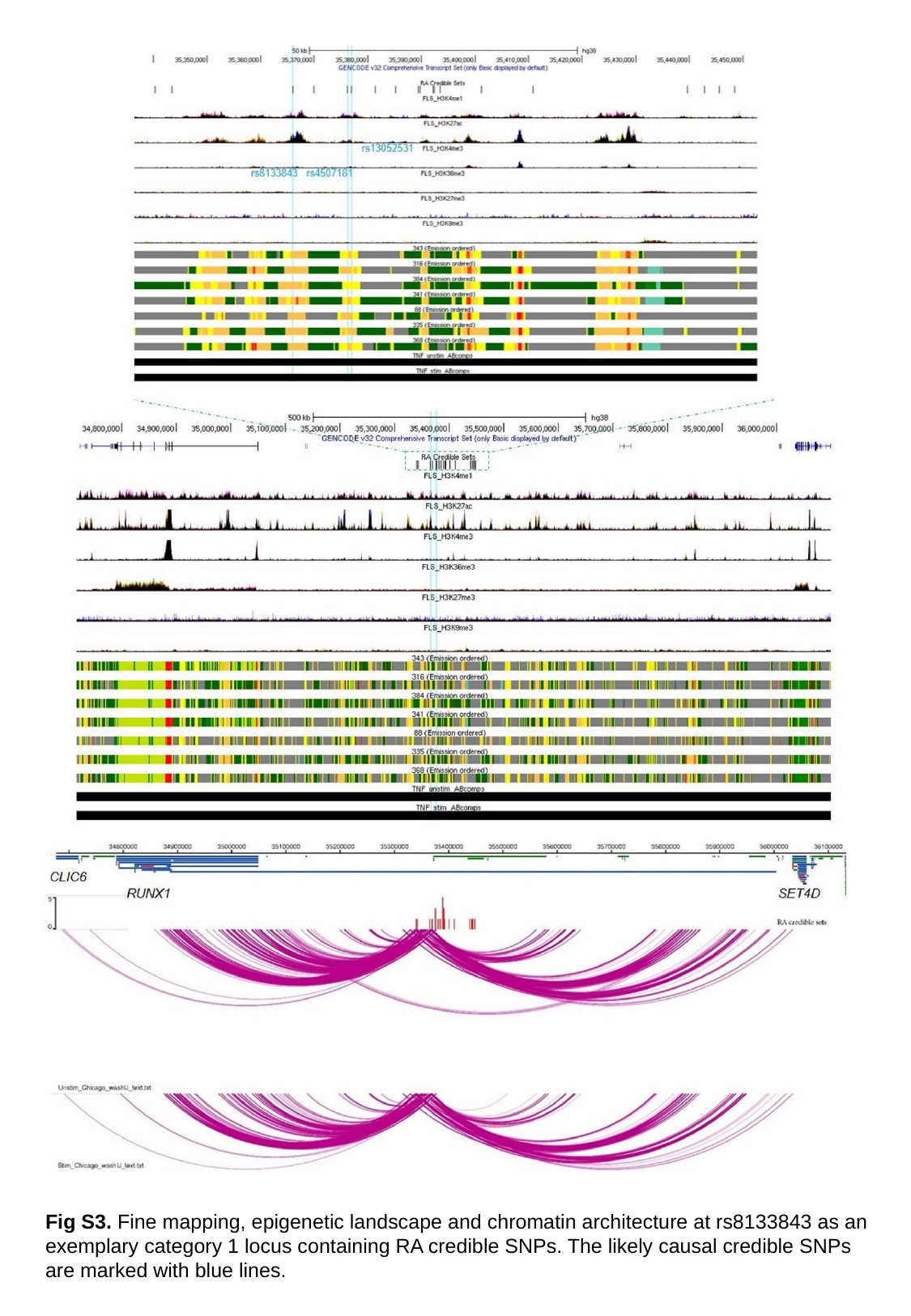

Fig S3. Fine mapping, epigenetic landscape and chromatin architecture at rs8133843 as an exemplary category 1 locus containing RA credible SNPs. The likely causal credible SNPs are marked with blue lines.

## Slide 4
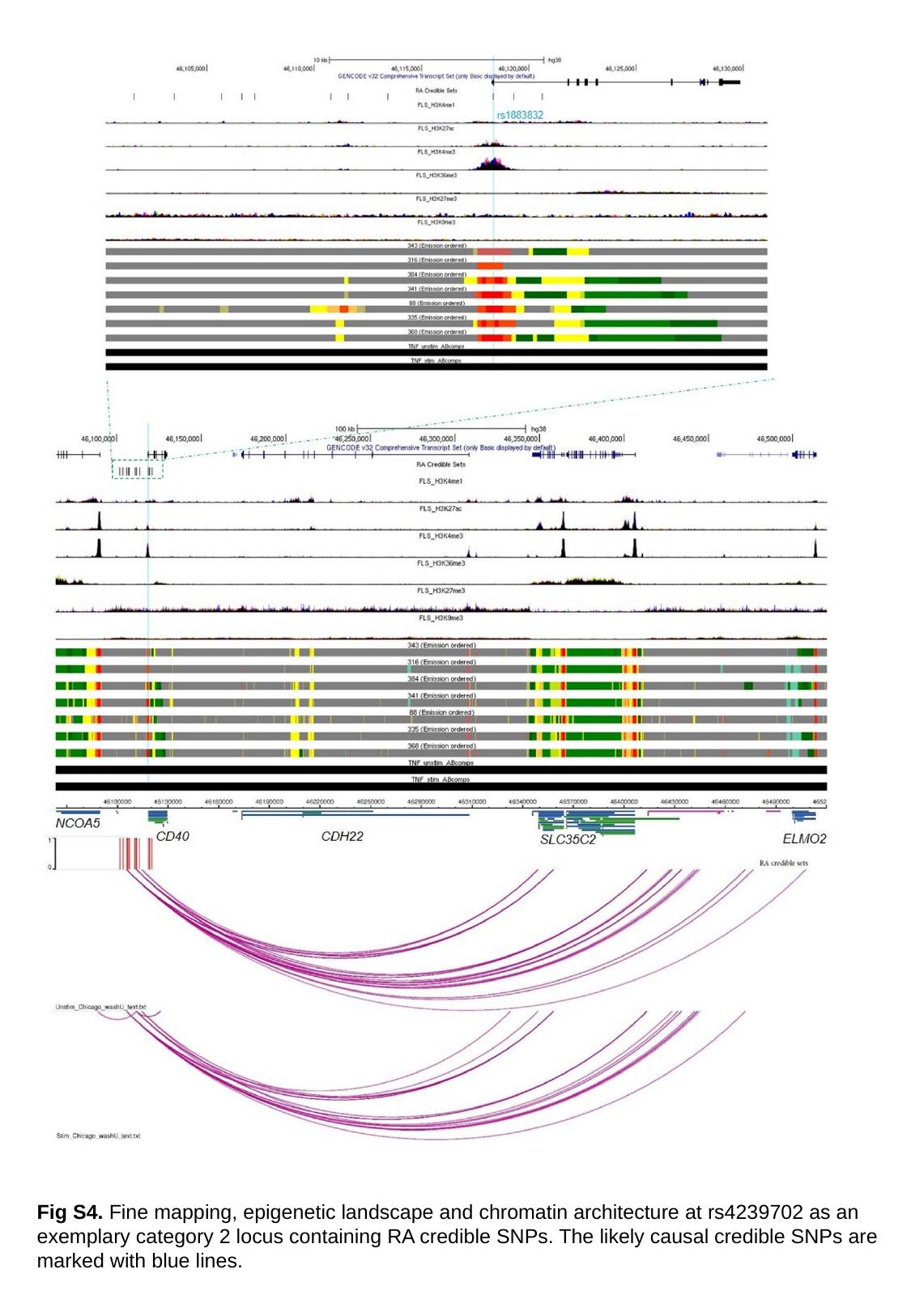

Fig S4. Fine mapping, epigenetic landscape and chromatin architecture at rs4239702 as an exemplary category 2 locus containing RA credible SNPs. The likely causal credible SNPs are marked with blue lines.

## Slide 5
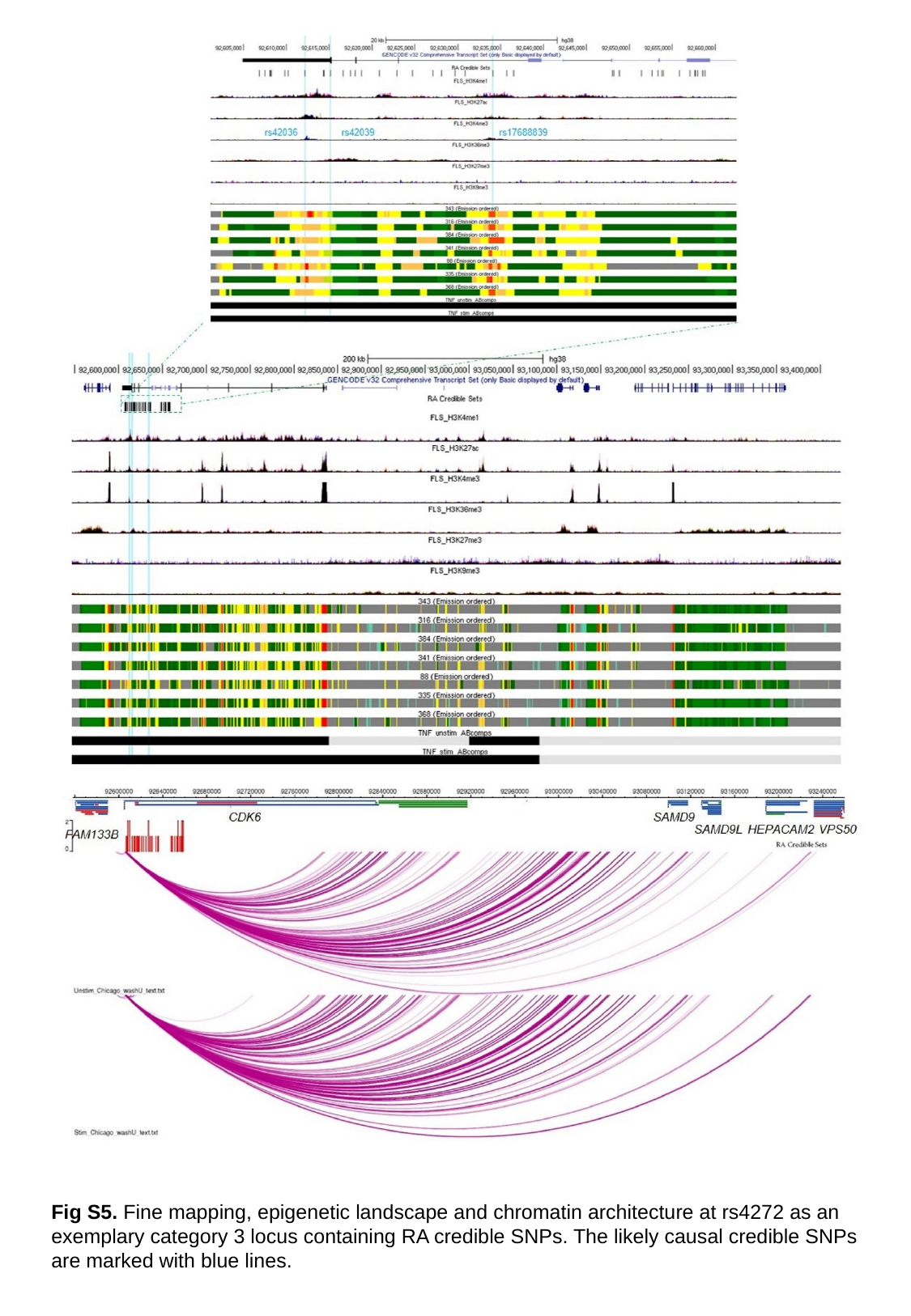

Fig S5. Fine mapping, epigenetic landscape and chromatin architecture at rs4272 as an exemplary category 3 locus containing RA credible SNPs. The likely causal credible SNPs are marked with blue lines.

## Slide 6
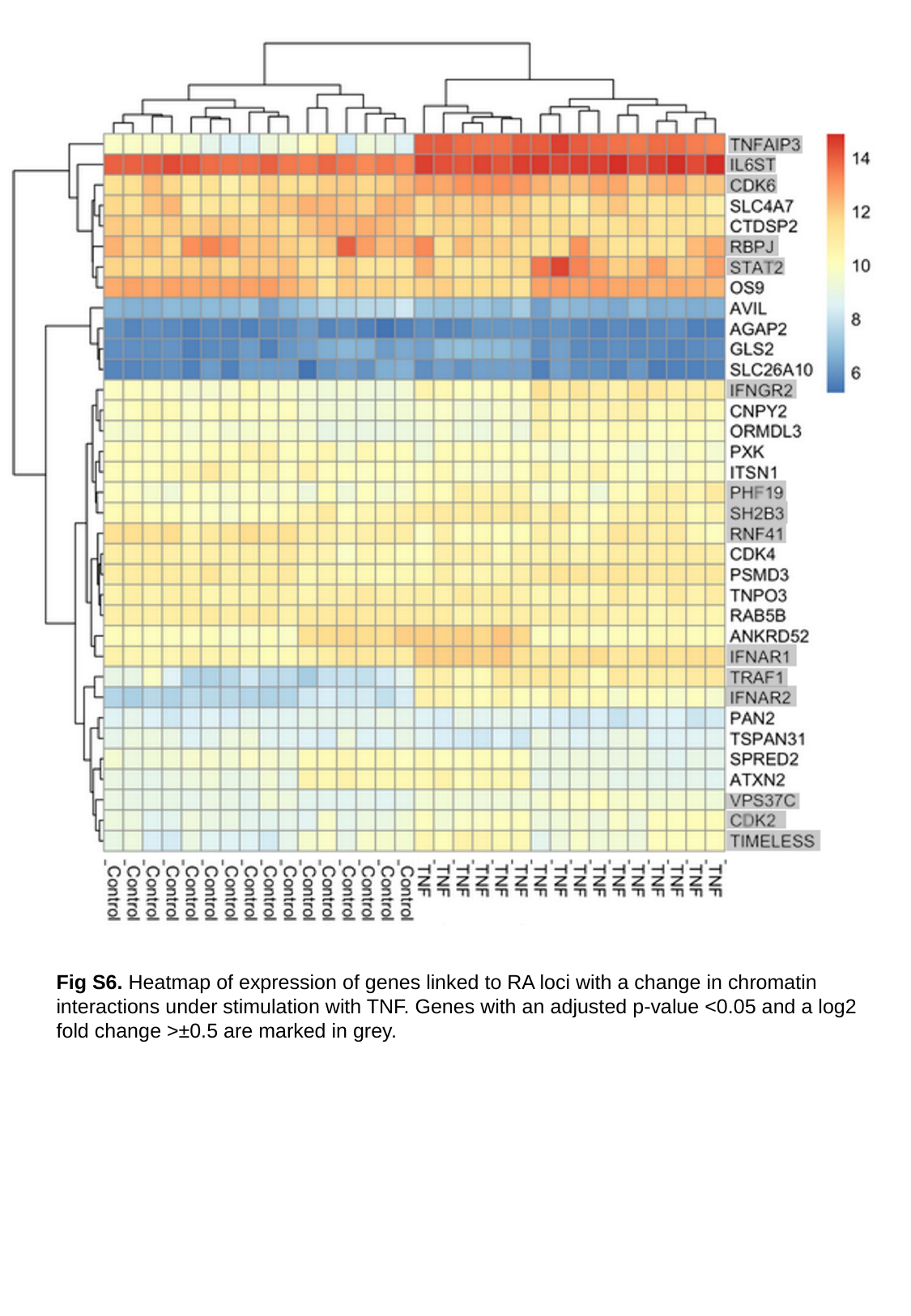

Fig S6. Heatmap of expression of genes linked to RA loci with a change in chromatin interactions under stimulation with TNF. Genes with an adjusted p-value <0.05 and a log2 fold change >±0.5 are marked in grey.

## Slide 7
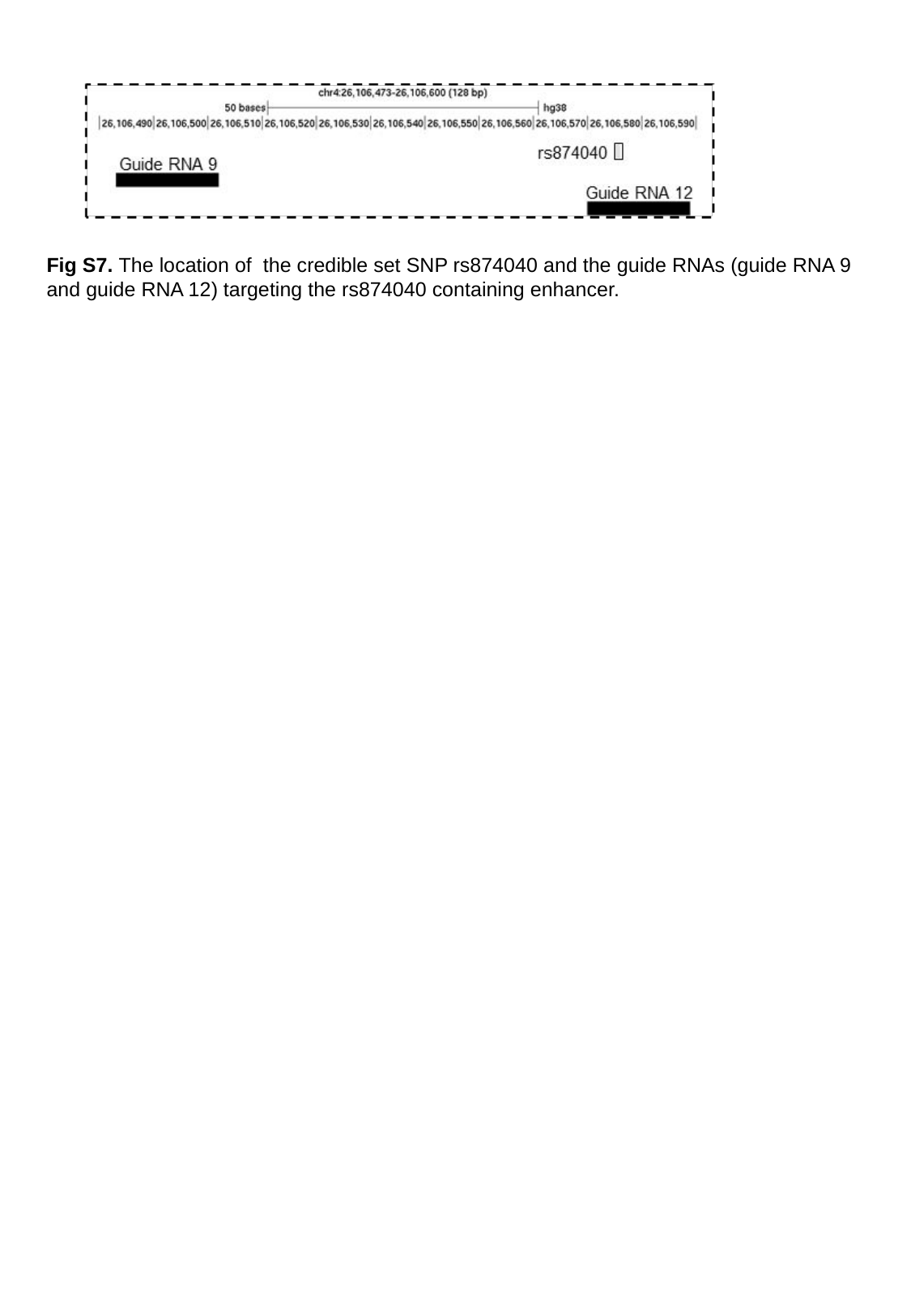

Fig S7. The location of the credible set SNP rs874040 and the guide RNAs (guide RNA 9 and guide RNA 12) targeting the rs874040 containing enhancer.
