# Supplementary material for: Functional genomics atlas of synovial fibroblasts defining rheumatoid arthritis heritability
Source: Genome Biol. 2021 Aug 25;22:247. doi: 10.1186/s13059-021-02460-6 (PMC8385949; doi:10.1186/s13059-021-02460-6)
Supplement: Supplementary file 9 — Additional file 9:. Review history [file 13059_2021_2460_MOESM9_ESM.docx]

Review History

**First round of review**

**Reviewer 1**

**Are you able to assess all statistics in the manuscript, including the appropriateness of statistical tests used?** There are no statistics in the manuscript.

**Comments to author:**

In the manuscript GBIO-D-21-00016, Ge et al reported multi-omic molecular profiling of synovial fibroblasts (FLS). The authors generated multidimensional datasets of RNA-seq, ChIP-seq, Hi-C and capture Hi-C in FLS cells derived from patients with rheumatoid arthritis (RA). By conducting integrative analysis, the authors identified putative causal variants, enhancers, genes, and cell types for RA. The authors showed that TNF stimulation of FLS altered TAD organization, chromatin state and the expression of putative causal genes. The authors also showed that risk variants could have joint-specific effects on target gene expression in RA FLS. However, the authors could have used more carefulness in data organization and interpretation, and data validation and integrative analysis can be improved.

Specific concerns:

1. The paper can be written/organized in a more neat way;

2. Based on Table S2, Hi-C/cHi-C, ATAC-seq and CAGE were all on RA cells, but no healthy cells as controls;

3. In Fig 1b, it would be nice to 1) clarify which tracks are from healthy cells or patient cells; and 2) include RNA-seq tracks;

4. One can hardly see anything from Fig 4b. It might be helpful if only focus on T, B and FLS cells in Fig 4b. The original Fig 4b can be moved to supplementary figures;

5. It is very puzzling that the authors employed CRISPRa (a gain of function assay) to validate enhancer activities (Fig 7b). It's probably true that any genomic loci might have shown moderate enhancer activity when VPR is tethered to. Loss of function assays (e.g., CRISPRi) should be more appropriate;

6. In Figs 7c, 7d and 7e, are the differences in expression statistically significant?

7. There is no Fig 7f and 7g;

8. Fig 8 was not cited/discussed in the main text.

**Reviewer 2**

**Are you able to assess all statistics in the manuscript, including the appropriateness of statistical tests used?** No, I do not feel adequately qualified to assess the statistics.

**Comments to author:**

In this manuscript, Ospelt's team perform a comprehensive integrated analysis of chromatin features (ChIP-seq of 6 histone marks, HiC, capture ATACseq) and gene expression (RNAseq) in FLS of genetically characterized RA patients. They also integrate these dat in B and T cells. The authors describe novel associations between genetic variants and functional defects in FLS of RA patients. The high variety and amount of data generated is of relevant interest for the understanding of RA molecular pathogenesis. Also, the integration analysis of the different data layers is original and interesting itself. However, there are some points that need to be addressed in order to confirm the authors' conclusions. There are also some minor issues that the authors should consider.

General format issues:

The manuscript is sometimes hard to follow:

*	There is also some degree of interspersing of discussion within the results section, which makes the text less fluid. Discussion should be placed within the discussion section.

*	Consistency in general terminology is lacking ("DNA activity" instead of "DNA accessibility").

Figures could be improved to show results more clearly:

*	Consistency in labelling between different figures is deficient (i.e. log fold-change should be labelled the same way throughout the article. "logFC", for instance).

*	I found excessive description of results in the text that could easily be placed within figures (percentages, p-values, etc. i.e. Figure 3d), making the text more readable and the figure more self-intelligible.

Specific issues:

*	In Figure 2 (section 1), although results are consistent, representation could be more clear. For instance, including a bar plot would make these results much easier to interpret.

*	Supplementary Figure 1: This plot is essentially flawed. The heatmap presented herein is scaled by column. Since here the authors are comparing between "Control" and "TNF" (in columns), data scaling should be performed between values of the same row (genes). Otherwise the changes in the expression are not appreciated at all.

*	In Section 2, authors never explain the discrepancies between "known" and "de novo" motif enrichment analysis. The differences found among the two approaches should at least be briefly discussed.

*	Figure 3: There are several issues with the data presented in this figure:

o	The usage of the same color palette for subfigures a and b (which actually refer to different types of data) induces wrong interpretations. Therefore, picking a different palette would help to avoid this issue.

o	Authors state that differential interaction intensity correlates with change in gene expression, although they do not provide a significance value for that correlation. A p-value derived from a correlation analysis (Pearson / Spearman) would be required (this could be done on absolute logFC of expression, for example).

o	Text labelling on the x axis is not clear. Indications on whether it indicates logFC(unstim/TNF) or logFC(TNF/unstim), or to which side is "more expressed in" each condition would be of great help.

*	Figure 4b, color scale is not labelled.

*	Section 4 requires a figure for explanation. A diagram -comparable to those found in other figures- is required to understand the pipeline followed by the authors to integrate and filter the results. The numbers stated in the text should appear in the diagram.

*	In Figure 5, it would be nice to see RNA-seq (to visualize expression differences) lanes included in the genome browser snapshots. Also, compartment A / B colors should be indicated with a color legend. Also, it is necessary to include information about statistical differences between conditions, at least for the differences stated for the text. For example, in Figure 5B, the authors state that there is a change in the accessibility of the IFN locus after stimulation, even though that is hard to see. An additional lane highlighting differentially accessible regions is required in the genome browser picture. Also, Figure 5a interesting locus is highlighted with a red arrow while there is no highlighting at Figure 5b. All those issues make Figure 5B especially hard to interpret.

*	Section 9 CRISPR-Cas9 experiment qPCR data lacks significance values (Figure 7b). Even though the results are interesting, the relatively slight effect in changes in expression (~30%) makes the result slightly dubious. Replication of the same experiment in at least another region would be appreciated and would strengthen the conclusion. Also, Figure 7a has a lot of extra information that is not informative, such as multiple lanes in the genome browser.

*	Figures 7f and 7g are included in the manuscript but lacking in the figures. Therefore, the last section is impossible to follow.

**Authors Response**

**Point-by-point responses to the reviewers’ comments:**

Reviewer 1:

The manuscript GBIO-D-21-00016, Ge et al reported multi-omic molecular profiling of synovial fibroblasts (FLS). The authors generated multidimensional datasets of RNA-seq, ChIP-seq, Hi-C and capture Hi-C in FLS cells derived from patients with rheumatoid arthritis (RA). By conducting integrative analysis, the authors identified putative causal variants, enhancers, genes, and cell types for RA. The authors showed that TNF stimulation of FLS altered TAD organization, chromatin state and the expression of putative causal genes. The authors also showed that risk variants could have joint-specific effects on target gene expression in RA FLS. However, the authors could have used more carefulness in data organization and interpretation, and data validation and integrative analysis can be improved.

Specific concerns:

1. The paper can be written/organized in a more neat way;

*Response: We have restructured the results section so that we feel it is easier to follow the flow of our experiments and results. In addition, we have revised the text to make it more fluent and easier to read. We also added an additional flowchart illustrating how we came to our credible set SNPs (now Supplementary Figure 1).*

2. Based on Table S2, Hi-C/cHi-C, ATAC-seq and CAGE were all on RA cells, but no healthy cells as controls;

*Response: That is correct. Unfortunately, healthy synovial fibroblasts were not available in the large number of cells needed for these experiments. Logistically and technically, it is very difficult to obtain FLS from healthy synovium. Their number in the starting material (biopsies) is lower and they proliferate less. This is the reason why most studies in the literature using FLS do not include healthy control samples. We think that, although the comparison between healthy, OA, and RA FLS would obviously be of interest, with the number of patient replicates we had available (n = 3), such a comparison would not provide robust results. Of course, we cannot exclude the possibility that there are differences in chromatin interactions and open chromatin between healthy and RA-FLS that we cannot detect in our study and that would affect our results. However, we also cannot rule out the possibility that some of the potential differences between RA FLS and healthy FLS are already present before the onset of the disease and that this is the reason why these genetic risk factors trigger the disease in some people but not in others. To address these research questions would require a different study design, which we are keeping in mind for the future. These points are now included in the discussion section of the revised manuscript.*

3. In Fig 1b, it would be nice to 1) clarify which tracks are from healthy cells or patient cells; and 2) include RNA-seq tracks;

*Response: We included the source of the tracks in the figure and the figure legend and added the RNA-seq tracks as the reviewer suggested.*

4. One can hardly see anything from Fig 4b. It might be helpful if only focus on T, B and FLS cells in Fig 4b. The original Fig 4b can be moved to supplementary figures;

*Response: We did as the reviewer suggested, simplified Fig. 4b and put the original figure as supplement. We absolutely agree that this improved the clarity of Fig. 4b.*

5. It is very puzzling that the authors employed CRISPRa (a gain of function assay) to validate enhancer activities (Fig 7b). It's probably true that any genomic loci might have shown moderate enhancer activity when VPR is tethered to. Loss of function assays (e.g., CRISPRi) should be more appropriate;

*Response: We absolutely understand the point made by the reviewer. Unfortunately, due to the current COVID-19 situation, we did not manage to gather the resources to establish new stable dCAS9-expressing FLS lines in the time frame given for the review (lack of cells, personnel, and consumables). With the resources we had available, we tried less complex approaches using transient transfections of FLS with CAS9 RNPs (reverse transfection and nucleofection protocols), but it was not possible to achieve sufficient transfection efficiency in the primary FLS to proceed. We then performed luciferase assays with luciferase promoter vectors containing oligonucleotides (31 bp) with the respective risk and non-risk variants in the middle and transfected them into a human fibroblast cell line (HT1080) to validate that the risk regions we identified indeed exhibit enhancer activity (now Fig. 7b).*

*We are very frustrated by this situation and can only refer the reviewer to the literature that supports the soundness of our experiments. Our protocol is based on several papers showing that CRISPR/dCas9-VPR is a valid tool for gene activation (Chavez, A., et al. Nat Methods 12, 326–328 (2015); Gilbert, et al. Cell, 154, 442-445 (2013); Perez-Pinera, al. Nat Methods 10, 973–976 (2013). However. Li et al just recently published a protocol to optimize activation of enhancer regions by CRISPR technology and compared their method with currently used activating protocols, showing that conventional activating CRISPR approaches often show only limited effects when targeting enhancers, as also seen in our dataset (Li, K., et al. Nat Commun 11, 485 (2020)).*

6. In Figs 7c, 7d and 7e, are the differences in expression statistically significant?

*Response: We now included statistical testing on the data in Figs 7c, 7d and 7e. However, with this low number of replicates, a p-value has to be interpreted with caution. We now also included this point in the discussion as a limitation of our study.*

7. There is no Fig 7f and 7g;

*Response: We are very sorry for this mistake; the mislabeling of the Figure panels is corrected in the revised manuscript. What was annotated as Fig 7f and 7g in the text was indeed Fig 8 a/b and c.*

8. Fig 8 was not cited/discussed in the main text.

*Response: We are very sorry for this mistake; the mislabeling of the Figure panels is corrected in the revised manuscript. What was annotated as Fig 7f and 7g in the text was indeed Fig 8 a/b and c.*

Reviewer 2:

In this manuscript, Ospelt's team perform a comprehensive integrated analysis of chromatin features (ChIP-seq of 6 histone marks, HiC, capture ATACseq) and gene expression (RNAseq) in FLS of genetically characterized RA patients. They also integrate these dat in B and T cells. The authors describe novel associations between genetic variants and functional defects in FLS of RA patients. The high variety and amount of data generated is of relevant interest for the understanding of RA molecular pathogenesis. Also, the integration analysis of the different data layers is original and interesting itself. However, there are some points that need to be addressed in order to confirm the authors' conclusions. There are also some minor issues that the authors should consider.

General format issues:

The manuscript is sometimes hard to follow:

*	There is also some degree of interspersing of discussion within the results section, which makes the text less fluid. Discussion should be placed within the discussion section.

*	Consistency in general terminology is lacking ("DNA activity" instead of "DNA accessibility").

*Response: We extensively revised the organization of the text and the terminology to make the manuscript easier to read.*

Figures could be improved to show results more clearly:

*	Consistency in labelling between different figures is deficient (i.e. log fold-change should be labelled the same way throughout the article. "logFC", for instance).

*Response: We revised the labelling to make the presentation of the data more consistent.*

*	I found excessive description of results in the text that could easily be placed within figures (percentages, p-values, etc. i.e. Figure 3d), making the text more readable and the figure more self-intelligible.

*Response: The text has been revised accordingly and more information has been added to the Figures.*

Specific issues:

*	In Figure 2 (section 1), although results are consistent, representation could be more clear. For instance, including a bar plot would make these results much easier to interpret.

*Response: We changed the presentation of the data in Figure 2 a,b,c according to the suggestion of the reviewer and they are now shown as bar plots.*

*	Supplementary Figure 1: This plot is essentially flawed. The heatmap presented herein is scaled by column. Since here the authors are comparing between "Control" and "TNF" (in columns), data scaling should be performed between values of the same row (genes). Otherwise the changes in the expression are not appreciated at all.

*Response: We were extensively discussing this point and agree that we have to clarify the used methodology. Supplementary figure 1 represents the normalized values of all observations (TNF + CTRL) per gene sorted by columns using hierarchical clustering. The normalization was performed as part of the standardized DESeq2 workflow applying the concept of variance stabilizing transformations (VST) (Tibshirani 1988; Huber et al. 2003; Anders and Huber 2010). VST normalizes per all observations (TNF + CTRL) per gene, so basically what the reviewer suggested. We adjusted the method section in our manuscript accordingly. We have also created the same heatmap with Zstacks. However, since we lose one dimension of information (expression level of the gene compared to the other genes), we would prefer to leave the heatmap as it was.*

*	In Section 2, authors never explain the discrepancies between "known" and "de novo" motif enrichment analysis. The differences found among the two approaches should at least be briefly discussed.

*Response: The differences between “known” and “de novo” motif enrichment analysis are mentioned in the results and the discussion of the revised manuscript.*

*	Figure 3: There are several issues with the data presented in this figure:

o	The usage of the same color palette for subfigures a and b (which actually refer to different types of data) induces wrong interpretations. Therefore, picking a different palette would help to avoid this issue.

o	Authors state that differential interaction intensity correlates with change in gene expression, although they do not provide a significance value for that correlation. A p-value derived from a correlation analysis (Pearson / Spearman) would be required (this could be done on absolute logFC of expression, for example).

o	Text labelling on the x axis is not clear. Indications on whether it indicates logFC(unstim/TNF) or logFC(TNF/unstim), or to which side is "more expressed in" each condition would be of great help.

*Response: The figure has been updated according to the suggestions of the reviewer with a different color palette and a better description of the x-axis. Due to a new version of ensemble and R the graph does look slightly different, but the outcome and statistical analysis, which is now included, remained the same.*

*	Figure 4b, color scale is not labelled.

*Response: Figure 4b has been modified to make more easy to read and the color scaling was adapted accordingly.*

*	Section 4 requires a figure for explanation. A diagram -comparable to those found in other figures- is required to understand the pipeline followed by the authors to integrate and filter the results. The numbers stated in the text should appear in the diagram.

*Response: We included a flowchart showing the different steps of the analysis and the number of risk regions and SNPs that resulted from each step (now Supplementary Figure 1).*

*	In Figure 5, it would be nice to see RNA-seq (to visualize expression differences) lanes included in the genome browser snapshots. Also, compartment A / B colors should be indicated with a color legend. Also, it is necessary to include information about statistical differences between conditions, at least for the differences stated for the text. For example, in Figure 5B, the authors state that there is a change in the accessibility of the IFN locus after stimulation, even though that is hard to see. An additional lane highlighting differentially accessible regions is required in the genome browser picture. Also, Figure 5a interesting locus is highlighted with a red arrow while there is no highlighting at Figure 5b. All those issues make Figure 5B especially hard to interpret.

*Response: We followed the suggestions of the reviewer to improve the figure. We added the RNA sequencing tracks and included a color legend for A/B compartments and an arrow highlighting the risk locus of interest. We agree with the reviewer that in Figure 5B differences in interactions after TNF stimulation are hard to see by eye, but all regions with significant changes in chromatin interactions as determined by DEseq2 are listed in Table 4. We now also refer to the results of the statistical analysis listed in table 4 in the Figure legend of Fig. 5.*

*	Section 9 CRISPR-Cas9 experiment qPCR data lacks significance values (Figure 7b). Even though the results are interesting, the relatively slight effect in changes in expression (~30%) makes the result slightly dubious. Replication of the same experiment in at least another region would be appreciated and would strengthen the conclusion. Also, Figure 7a has a lot of extra information that is not informative, such as multiple lanes in the genome browser.

*Response: We absolutely agree that additional analysis of the functional role in a different region would strengthen the validity of our experiments. Unfortunately, due to the current COVID-19 situation, we did not manage to gather the resources to establish new stable dCAS9-expressing FLS lines in the time frame given for the review (lack of cells, personnel, and consumables). With the resources we had available, we tried less complex approaches using transient transfection of FLS with CAS9 RNPs (reverse transfection and nucleofection protocols), but it was not possible to achieve sufficient transfection efficiency in the primary FLS to proceed. We then performed luciferase assays with luciferase promoter vectors containing oligonucleotides (31 bp) with the respective risk and non-risk variants in the middle and transfected them into a human fibroblast cell line (HT1080) to validate that the risk regions we identified indeed exhibit enhancer activity (now Fig. 7b).*

*We are very frustrated by this situation and can only refer the reviewer to the literature that supports the soundness of our experiments and shows that a modest effect has been previously described with this approach. Our protocol is based on several papers showing that CRISPR/dCas9-VPR is a valid tool for gene activation (Chavez, A., et al. Nat Methods 12, 326–328 (2015); Gilbert, et al. Cell, 154, 442-445 (2013); Perez-Pinera, al. Nat Methods 10, 973–976 (2013). However. Li et al just recently published a protocol to optimize activation of enhancer regions by CRISPR technology and compared their method with currently used activating protocols, showing that conventional activating CRISPR approaches often show only limited effects when targeting enhancers as seen in our dataset (Li, K., et al. Nat Commun 11, 485 (2020)).*

*We changed Fig. 7a to make it easier to read.*

*	Figures 7f and 7g are included in the manuscript but lacking in the figures. Therefore, the last section is impossible to follow.

*Response: We are really sorry for this mistake, which is corrected in the new manuscript. Fig.7f and Fig7g were indeed Fig 8a/b and Fig8c.*

**Second round of review**

**Reviewer 1**

Although the revised manuscript is much improved, there are still some concerns regarding the quality and interpretation of the data.

Major concerns:

1. Results in Fig 5. The authors stated that “TNF stimulation of FLS induced dynamic changes in chromatin interactions at this locus and increased the expression of IFNAR2, IFNAR1, and IFNGR2 (Supplementary Figure 1, Table 42, Fig. 5b). Additionally, chromatin accessibility in the region of IFNAR2 changed (from an inactive B to an active A compartment) in stimulated FLS (Fig. 5b).” But based on Fig 5b, 1) The expression of these three genes are NOT increased but rather slightly decreased upon stimulation; 2) the three genes and the SNPs are located in the A compartment with or without stimulation; and 3) there is no apparent difference in both ATAC-seq and Capture Hi-C signals after stimulation.

2. Results in Fig 7.

1)	In Fig 7b, the three disease-associated SNPs has no positive influence on enhancer activity in the luciferase reporter assays;

2)	The differences are so moderate in Fig 7c, 7d and 7e.

**Reviewer 2**

I am satisfied with the revisions. It is a pity that the authors did not manage to establish new stable dCAS9-expressing FLS lines in the time frame given for the review, but all the other issues have been addressed.

**Authors Response**

**Point-by-point responses to the reviewers’ comments:**

**Reviewer 1**

Although the revised manuscript is much improved, there are still some concerns regarding the quality and interpretation of the data.

Major concerns:

1. Results in Fig 5. The authors stated that “TNF stimulation of FLS induced dynamic changes in chromatin interactions at this locus and increased the expression of IFNAR2, IFNAR1, and IFNGR2 (Supplementary Figure 1, Table 42, Fig. 5b). Additionally, chromatin accessibility in the region of IFNAR2 changed (from an inactive B to an active A compartment) in stimulated FLS (Fig. 5b).” But based on Fig 5b, 1) The expression of these three genes are NOT increased but rather slightly decreased upon stimulation; 2) the three genes and the SNPs are located in the A compartment with or without stimulation; and 3) there is no apparent difference in both ATAC-seq and Capture Hi-C signals after stimulation.

*Response: I agree that in the visualization of the data it is difficult to estimate differences from eye from the examples shown, but that is why we apply statistics on all the samples. 1) The analysis in table 2 clearly shows that these genes are induced, 2) we only say that the region of IFNAR2 changes compartment activity, not ‘the three genes and the SNPs’, 3) again, we performed statistical analysis as described in the methods on all the datasets and there was a statistically significant difference, even though this might not be seen from eye.*

2. Results in Fig 7.

1)	In Fig 7b, the three disease-associated SNPs has no positive influence on enhancer activity in the luciferase reporter assays;

*Response: The reviewer might have expected a bigger effect, but the effect was significant and is in absolute concordance to the extent of effects that are expected in these kind of assays from the literature.*

2)	The differences are so moderate in Fig 7c, 7d and 7e.

*Response: In the reviewer’s opinion these differences are moderate, but many eQTL studies show much smaller differences, which become statistically significant because of the bigger sample size. With synovial tissue samples it is not possible to reach these high numbers and we also clearly address this limitations of the study.*

*All in all, these are also just examples of genes we give, because they are well known and might be of interest to the reader. The criticized parts are for sure not the key messages of the paper, which is the comprehensive analysis of genetic risk loci in RA that might be functional in FLS. I just cannot believe that Genome Biology would reject a paper based on these comments which seem very subjective, mainly concern the visualization of some exemplary data and are not supported by what is shown in similar studies in this topic.*
